# Molecular mechanisms of pancreatic cancer liver metastasis: the role of PAK2

**DOI:** 10.3389/fimmu.2024.1347683

**Published:** 2024-01-26

**Authors:** Hao Yang, Zhongyi Li, Shiqi Zhu, Wenxia Wang, Jing Zhang, Dongxu Zhao, Man Zhang, Wenxin Zhu, Wei Xu, Chunfang Xu

**Affiliations:** ^1^ Department of Gastroenterology, The First Affiliated Hospital of Soochow University, Suzhou, Jiangsu, China; ^2^ Department of General, Visceral, and Transplant Surgery, Ludwig-Maximilians-University Munich, Munich, Germany; ^3^ Department of General Medicine, Zhongshan Hospital Fudan University, Shanghai, China; ^4^ Division of Basic Biomedical Sciences, The University of South Dakota Sanford School of Medicine, Vermillion, SD, United States; ^5^ Department of Interventional Radiology, The Affiliated Changshu Hospital of Nantong University, Changshu No. 2 People‘s Hospital, Changshu, Jiangsu, China; ^6^ Department of Emergency Medicine, The Affiliated Hospital of Xuzhou Medical University, Xuzhou, Jiangsu, China; ^7^ Department of Gastroenterology, Kunshan Third People’s Hospital, Suzhou, Jiangsu, China

**Keywords:** pancreatic cancer, liver metastasis, machine learning, single cell sequencing, bioinformatics

## Abstract

**Background:**

Pancreatic cancer remains an extremely malignant digestive tract tumor, posing a significant global public health burden. Patients with pancreatic cancer, once metastasis occurs, lose all hope of cure, and prognosis is extremely poor. It is important to investigate liver metastasis of Pancreatic cancer in depth, not just because it is the most common form of metastasis in pancreatic cancer, but also because it is crucial for treatment planning and prognosis assessment. This study aims to delve into the mechanisms of pancreatic cancer liver metastasis, with the goal of providing crucial scientific groundwork for the development of future treatment methods and drugs.

**Methods:**

We explored the mechanisms of pancreatic cancer liver metastasis using single-cell sequencing data (GSE155698 and GSE154778) and bulk data (GSE71729, GSE19279, TCGA-PAAD). Initially, Seurat package was employed for single-cell data processing to obtain expression matrices for primary pancreatic cancer lesions and liver metastatic lesions. Subsequently, high-dimensional weighted gene co-expression network analysis (hdWGCNA) was used to identify genes associated with liver metastasis. Machine learning algorithms and COX regression models were employed to further screen genes related to patient prognosis. Informed by both biological understanding and the outcomes of algorithms, we meticulously identified the ultimate set of liver metastasis-related gene (LRG). In the study of LRG genes, various databases were utilized to validate their association with pancreatic cancer liver metastasis. In order to analyze the effects of these agents on tumor microenvironment, we conducted an in-depth analysis, including changes in signaling pathways (GSVA), cell differentiation (pseudo-temporal analysis), cell communication networks (cell communication analysis), and downstream transcription factors (transcription factor activity prediction). Additionally, drug sensitivity analysis and metabolic analysis were performed to reveal the effects of LRG on gemcitabine resistance and metabolic pathways. Finally, functional experiments were conducted by silencing the expression of LRG in PANC-1 and Bx-PC-3 cells to validate its influence to proliferation and invasiveness on PANC-1 and Bx-PC-3 cells.

**Results:**

Through a series of algorithmic filters, we identified PAK2 as a key gene promoting pancreatic cancer liver metastasis. GSVA analysis elucidated the activation of the TGF-beta signaling pathway by PAK2 to promote the occurrence of liver metastasis. Pseudo-temporal analysis revealed a significant correlation between PAK2 expression and the lower differentiation status of pancreatic cancer cells. Cell communication analysis revealed that overexpression of PAK2 promotes communication between cancer cells and the tumor microenvironment. Transcription factor activity prediction displayed the transcription factor network regulated by PAK2. Drug sensitivity analysis and metabolic analysis revealed the impact of PAK2 on gemcitabine resistance and metabolic pathways. CCK8 experiments showed that silencing PAK2 led to a decrease in the proliferative capacity of pancreatic cancer cells and scratch experiments demonstrated that low expression of PAK2 decreased invasion capability in pancreatic cancer cells. Flow cytometry reveals that PAK2 significantly inhibited apoptosis in pancreatic cancer cell lines. Molecules related to the TGF-beta pathway decreased with the inhibition of PAK2, and there were corresponding significant changes in molecules associated with EMT.

**Conclusion:**

PAK2 facilitated the angiogenic potential of cancer cells and promotes the epithelial-mesenchymal transition process by activating the TGF-beta signaling pathway. Simultaneously, it decreased the differentiation level of cancer cells, consequently enhancing their malignancy. Additionally, PAK2 fostered communication between cancer cells and the tumor microenvironment, augments cancer cell chemoresistance, and modulates energy metabolism pathways. In summary, PAK2 emerged as a pivotal gene orchestrating pancreatic cancer liver metastasis. Intervening in the expression of PAK2 may offer a promising therapeutic strategy for preventing liver metastasis of pancreatic cancer and improving its prognosis.

## 1 Introduction

Pancreatic cancer, a gastrointestinal malignancy with inconspicuous symptoms, presents a significant diagnostic challenge and suboptimal treatment outcomes. The mechanisms underlying its occurrence and metastasis remain largely unknown. The lethality of pancreatic cancer is attributed to three primary factors: a lack of early detection methods, early metastasis leading to a loss of surgical opportunities, and a complex tumor immune microenvironment (1). About 80% of pancreatic cancer patients miss the opportunity for surgery upon diagnosis, leading to a 5-year survival rate of merely 6-8%. Even with surgical intervention, the 5-year survival rate experiences only a modest increase to 20% (2). Therefore, research on pancreatic cancer, especially its pathogenesis, early diagnostic markers, and novel therapeutic strategies, holds significant clinical significance.

Pancreatic cancer lacks specific early symptoms, corresponding tumor markers, and imaging features, making early detection and diagnosis challenging. The elevated invasiveness of pancreatic cancer cells contributes to frequent instances of local infiltration and metastasis. Pancreatic cancer metastasis is a multifaceted process that encompasses the interplay between tumor cells and the microenvironment, coupled with the activation of diverse signaling pathways. Despite numerous studies revealing the mechanisms of pancreatic cancer over past decade, seemingly beneficial for diagnosis and treatment, it is acknowledged that pancreatic cancer exhibits significant heterogeneity among different tumors (3). The flourishing development of sequencing technologies, from early gene chips to current high-throughput sequencing, enables in-depth transcriptomic studies of pancreatic cancer. Faced with the genetic heterogeneity of pancreatic cancer, researchers such as Moffit (4), Collisson (5), and Bailey (6) have proposed distinct molecular subtypes, providing a basis for understanding and treating pancreatic cancer. The advancement of single-cell sequencing technology allows for cell-level analysis, identifying the relationships between different cell subgroups and the microenvironment. Moreover, investigating the interplay between cells and the microenvironment offers deeper insights into the biological behavior of tumors and the mechanisms of treatment resistance. These research findings not only enhance our understanding of the complexity of pancreatic cancer but also provide potential avenues for developing more effective personalized treatment strategies.

This study has various strengths, leveraging the respective advantages of single-cell transcriptomics and traditional bulk sequencing technologies, enabling us to achieve a more comprehensive breakthrough in cancer research. Firstly, by harnessing the precision of single-cell transcriptomics in conjunction with the large-scale processing capability of traditional bulk sequencing technology, we successfully identified a series of genes stably expressed in cancer cells. These genes not only exhibit excellent performance in diagnosis but also demonstrate significant efficacy in predicting patient prognosis. Diverging from studies solely focused on identifying diagnostic or prognostic markers, we conducted in-depth analyses of the mechanistic actions of key genes, covering nearly all mechanisms that promote tumor progression. This approach added systematic depth to our research, providing a more thorough understanding of the multifaceted factors influencing cancer development. Lastly, our research extended beyond theoretical analysis, incorporating experimental validation to robustly support our analytical findings. This experimental validation not only enhanced the reliability of our study but also established a solid foundation for the future application of these discoveries in clinical practice.

## 2 Methods

### 2.1 Pancreatic cancer liver metastasis single-cell sequencing data and bulk data download

The single-cell sequencing data for human pancreatic cancer tissues utilized in this study were sourced from the Gene Expression Omnibus (GEO) database under accession numbers GSE155698 and GSE154778. The GSE155698 dataset comprised sequencing information from 20 tissues, encompassing 17 pancreatic cancer tissues and 3 normal pancreatic tissues. The original FASTQ files of the GSE155698 dataset were pre-processed by the submitter using Cellranger (version 3.0.0) with default settings, and the initial expected cell count was set at 10,000. In all instances, the alignment was conducted using the hg19 reference provided with the cellranger software. The GSE154778 dataset was sequenced at an approximate depth of 50,000 reads per cell. The authors constructed an expression matrix using CellRanger (10x Genomics) and filtered out low-quality barcodes (cells). Additionally, cells with small library sizes (<1000 UMI) or expressing fewer genes were excluded.

Bulk sequencing data were retrieved from the GEO and The Cancer Genome Atlas (TCGA) databases using the search terms “pancreatic cancer” and “liver metastasis”. Two datasets, namely GSE71729 (comprising 145 primary lesions and 25 liver metastatic lesions) and GSE19279 (consisting of 4 primary lesions and 5 liver metastatic lesions), were identified for further analysis. Furthermore, TCGA data were downloaded using the search terms “pancreas”, “TCGA”, “TCGA-PAAD”, “transcriptome profiling”, and “Gene Expression Quantification”, resulting in 183 sequenced pancreatic cancer tissues. Subsequently, samples without survival information and normal samples were excluded, resulting in a final dataset of 172 samples containing prognostic information. The sequencing results of gemcitabine-resistant cell lines were derived from the GSE140077 dataset. The original dataset comprised 12 sequencing data, with 3 groups of BxPC-3 resistant strains and 3 groups of CFPAC-1 resistant strains considered as the experimental group. Meanwhile, 3 groups of wild-type BxPC-3 and 3 groups of wild-type CFPAC-1 were designated as the control group.

### 2.2 Methods for processing and visualization of single-cell sequencing data

The preliminary processing of the single-cell sequencing data was performed using the Seurat package. To prevent sequencing errors from affecting subsequent analyses, we implemented a two-step filtering process to remove low-quality cells and abnormal data such as non-single cells. The CreateSeuratObject function was employed to read and preliminarily filter all data. Cells and genes were retained based on specific criteria: a gene had to be expressed in at least 3 cells, and a cell had to express at least 250 genes. Any cells or genes not meeting these criteria were removed. After reading the data, we calculated the proportions of mitochondrial genes and rRNA genes in the dataset. The final filtering criteria were as follows: (1) Exclude cells with gene expression counts less than 500 or greater than 6000; (2) Ensure each cell’s UMI count was greater than 1000, and excluded cells with the top 3% of UMI count values; (3) The proportion of mitochondrial gene expression in each cell relative to the total genes should be less than 35%, and cells with the top 2% of mitochondrial gene expression were excluded; (4) Calculate the proportion of rRNA expression relative to the total genes and exclude the bottom 1% and top 1% of cells based on this proportion; (5) RNA count should be greater than 1000, and cells with the top 3% of RNA count values were excluded.

Subsequently, the Seurat dataset underwent further processing using the harmony package. First, the process included applying the NormalizeData function to normalize the single-cell matrix. Next, the FindVariableFeatures function was used for feature selection, selecting 2000 variable features using the “vst” method. Following this, the single-cell matrix underwent data scaling and principal component analysis (PCA) using the ScaleData and RunPCA functions, setting the npcs parameter to 30. Then, the RunHarmony function was applied, running the Harmony algorithm with “orig.ident” as the adjustment variable and generating a convergence plot. Finally, based on the results of Harmony, the RunUMAP function was used for Uniform Manifold Approximation and Projection (UMAP) dimensionality reduction, setting the top 30 principal components, and the FindNeighbors and FindClusters functions were employed for neighborhood search and cluster analysis to elucidate the data’s structure. Ultimately, cluster labels for the samples were obtained using the identity function.

### 2.3 Identifying liver metastasis-associated genes in pancreatic cancer single-cell sequencing

We employed the hdWGCNA package to identify pivotal module genes involved in pancreatic cancer liver metastasis. Firstly, we employed the SetupForWGCNA function to construct an expression matrix from the Seurat object. Subsequently, using the MetacellsByGroups function, we built averaged “metacells” based on cell types and cell sources (with 25 nearest neighbor cells and a maximum shared cell count of 10 between two metacells). Following this, we normalized and standardized the metacell matrix through various means. We applied dimensionality reduction to the metacell matrix using both PCA and the harmony algorithm. The UMAP algorithm was then used for projection. Next, we used the SetDatExpr function to set the expression matrix and the TestSoftPowers function to calculate the topological indicators of the network, assisting in the selection of the optimal soft threshold. Ultimately, the ConstructNetwork function was utilized to establish a co-expression network, and the ModuleEigengenes function was used to compute the “eigenvectors” of the modules to obtain module feature genes.

Following the acquisition of module genes, we conducted differential analysis using the FindMarkers function to pinpoint genes significantly altered during pancreatic cancer liver metastasis, applying a significance threshold of P<0.05. The overlap of genes from both analyses yielded the key genes involved in the pancreatic cancer liver metastasis process.

### 2.4 Further screening of liver metastasis-associated genes in bulk sequencing data

A univariate COX regression model was applied to delve deeper into genes associated with pancreatic cancer liver metastasis and to further screen molecular factors closely linked to patient prognosis. Various machine learning algorithms were employed for the initial screening of liver metastasis-associated genes. Subsequently, another round of screening was performed using multiple machine learning algorithms for prognosis-related liver metastasis-associated genes.

Initially, the Least Absolute Shrinkage and Selection Operator (LASSO) algorithm, utilizing the glmnet package, was employed. LASSO fits a generalized linear model, incorporating variable selection and complexity adjustment to enhance the model’s generalization ability. Subsequently, the Support Vector Machine Recursive Feature Elimination (SVM-RFE) algorithm, based on the e1071 package, was utilized. SVM-RFE, a sequence backward selection algorithm relying on support vector machines (SVM), optimized gene selection by extracting features through the maximum margin principle for two-class data. Finally, the Random Forest (RF) algorithm, employing the randomForest package, was applied. Random Forest calculated the average contribution of each feature in every decision tree within the random forest, effectively ranking features based on their contributions. The final step involved taking the intersection of genes selected by LASSO regression, SVM-RFE, and Random Forest. The top 15 genes from each method were considered.

The expression trends and prognostic implications of the intersected genes were then examined for final selection. This multi-tiered algorithmic selection and ranking approach facilitated a comprehensive and accurate exploration of genes closely associated with pancreatic cancer liver metastasis.

Furthermore, gene validation was conducted in various databases. In the GSE71729 and GSE19279 datasets, we observed the expression levels of genes in primary lesions and metastatic groups. The diagnostic efficiency of the genes for pancreatic cancer liver metastasis was identified through Receiver Operating Characteristic (ROC) analysis. Additionally, the roles of genes in pancreatic cancer occurrence were further analyzed in the above datasets, and protein-level validation of the genes encoded by these genes in pancreatic cancer/normal pancreatic tissues was performed in the human protein atlas.

### 2.5 Unveiling the impact of LRG on pathway activation in pancreatic cancer cells

Gene Set Variation Analysis (GSVA) was employed to identify pathway changes in pancreatic cancer cells induced by LRG. GSVA calculated the cumulative score of genes within a gene set, transforming samples into a metric of gene set activity, allowing us to assess differences in gene set activity across samples. Reference gene sets were obtained from the Kyoto Encyclopedia of Genes and Genomes (KEGG) database. The impact of LRG on signaling pathways in pancreatic cancer cells was evaluated separately in single-cell and bulk data. In single-cell data, our initial assessment focused on changes in signaling pathways in cancer cells from both primary pancreatic cancer lesions and liver metastatic lesions. Additionally, we combined all datasets, extracted cancer cells, and based on LRG expression, divided all cancer cells into high LRG expression cells and low LRG expression cells. Finally, we analyzed the differences in signaling pathways between these two cell types.

In transcriptomic data, all samples were categorized into high LRG expression and low LRG expression groups based on the median relative expression of LRG. GSVA was subsequently employed to analyze pathway differences between these two groups. The intersection of pathway results from these three analyses was considered the key pathways through which LRG primarily influences pancreatic cancer liver metastasis.

### 2.6 Assessing the impact of LRG on the differentiation level of pancreatic cancer cells

To investigate the impact of LRG on the differentiation level of pancreatic cancer cells, we employed a combination of various algorithms and datasets. Firstly, pseudotime analysis was conducted on single-cell data using the monocle2 package. After estimating size factors and dispersions, the detectGenes function was used to filter out low-quality cells by setting an expression threshold (0.1). Subsequently, we selected clusters of the top 200 differentially expressed genes and applied dimensionality reduction using the DDRTree method in the reduceDimension function. We then calculated developmental time, inferred trajectories, and ranked cells based on the pseudotime. The results were visualized using a tree plot. During trajectory inference, the beam statistical method was applied to the pseudotime-sorted cell data and specified nodes. This analysis calculated the contribution of genes during cell development and differentiation, ranking and outputting all key genes based on their contribution values. These genes played crucial roles in the process of cell development and differentiation. By observing the contribution values and P-values of LRG, we could determine if LRG played a role in the differentiation of pancreatic cancer cells. Additionally, pseudotime analysis was also performed using the monocle3 package. Single-cell data were stored in a SingleCellExperiment object, and a Monocle3 object was created. The reduceDimension function in Monocle3 was then used for dimensionality reduction with UMAP-learned t-SNE, and the orderCells function was applied to rank cells based on pseudotime. Lastly, the Monocle3 method was used to estimate and calculate cell development time. The graph_test function executed differential network analysis to detect differences in gene networks in single-cell data. The Moran index and P-values were crucial parameters to assess whether genes influenced the differentiation level. Finally, validation was performed in TCGA data. We extracted the Grade grouping from TCGA clinical data, observed gene expression in each group, conducted statistical tests using a logistic regression model, and visualized the results with a box plot.

### 2.7 Exploring changes in the communication network of pancreatic cancer cells with high LRG expression in the tumor microenvironment

We utilized the CellChat package to elucidate the influence of cells with high LRG expression on cell signaling communication in the tumor microenvironment compared to cells with low LRG expression. To achieve this, we utilized the Cell Communication Analysis method for inferring and analyzing cell-cell interaction networks. To start, within a cellular group, we identified ligands or receptors that exhibited overexpression and then integrated gene expression data into a protein-protein interaction (PPI) network. Once the overexpressed ligands or receptors were pinpointed, we could discern the interactions among them. Following this, we calculated the communication probability associated with ligand-receptor interactions specific to each signaling pathway, thereby deducing the pathway-level communication probability. By quantifying either the number of connections or the comprehensive communication probability, we computed the consolidated communication network among cells. In the end, we established a cellular communication network that integrated details about cell ligand-receptor interactions and communication at the pathway level. This approach provided a more profound understanding of how pancreatic cancer cells with elevated LRG expression regulated communication networks within the tumor microenvironment. The establishment of this cell communication network helped reveal dynamic changes and interactions in signal transmission between cells with high LRG expression and other cells.

### 2.8 Identification of downstream transcription factors regulated by LRG

The prediction of transcription factor (TF) activity was based on the “DoRothEA” package. DoRothEA is a gene regulatory network that encompasses signed interactions between TF and their target genes. After categorizing cancer cells, we accessed human regulatory element information from the DoRothEA database, filtering out regulatory elements with high confidence levels (A, B, C grades). Afterward, we applied the Viper (Virtual Inference of Protein-activity by Enriched Regulon analysis) algorithm to compute the activity scores of regulatory elements in cells. These scores reflected the activation levels of gene regulatory networks in various cell types. Next, we switched to the data of DoRothEA regulatory elements and performed cell standardization, PCA dimensionality reduction, neighbor search, cluster analysis, and UMAP dimensionality reduction. Using the FindAllMarkers function, we identified genes with significantly differential expression in different cell clusters. Lastly, we aggregated the Viper scores, computed the mean and standard deviation of each regulatory element across different cell types, and created a heatmap illustrating the activity levels of regulatory elements in various cell types using the pheatmap package.

### 2.9 Correlation analysis of LRG and gemcitabine resistance

We categorized TCGA patient samples into low and high LRG expression groups based on the median LRG expression. Subsequently, we conducted an analysis of gemcitabine drug sensitivity. The drug sensitivity analysis was carried out using the R package “pRRophetic,” developed by Paul Geeleher and colleagues in 2014. This package utilized the GDSC cell line expression profile and TCGA gene expression profile, constructing a ridge regression model to predict the half-maximal inhibitory concentration (IC50), which corresponded to the drug concentration at which the ratio of apoptotic cells to total cells is 50%. The results of the drug sensitivity analysis were visualized through box plots, illustrating the distribution of IC50 values among different samples. Additionally, correlation analysis was performed to reveal the relationship between LRG expression levels and IC50 values. Additionally, we conducted an analysis of LRG-mediated gemcitabine resistance in single-cell data. The gemcitabine resistance gene set was obtained from the GSE140077 dataset. We used differential analysis and Weighted Gene Co-expression Network Analysis (WGCNA) to identify gemcitabine resistance genes in pancreatic cancer. To identify the enrichment of gemcitabine resistance genes in the cells of the pancreatic cancer microenvironment, we performed enrichment analysis on the single-cell sequencing data. Gene set enrichment analysis in single-cell sequencing data was conducted using the “irGSEA” package, which integrated four algorithms: “AUCell”, “UCell”, “singscore”, and “ssgsea”. AUCell calculated the area under the curve (AUC) to assess whether a subset of the input gene set was enriched in the expression genes of each cell, and its ranking method was based on AUC scores. UCell scoring was based on gene ranking, demonstrating robustness to dataset size and heterogeneity. Singscore was a rank-based measurement of the degree of gene set enrichment in a single sample. Single-Sample Gene Set Enrichment Analysis (ssGSEA), a single-sample GSEA, computed the enrichment score of key gene sets in each cell. Following this, the Wilcoxon test was applied to identify differentially enriched gene sets in the enrichment score matrix, with a filter criterion of a corrected P-value less than 0.05. Then, the rank aggregation algorithm in the RobustRankAggreg package was used to comprehensively evaluate the results of differential analysis, filtering out significantly enriched gene sets in most gene set enrichment analysis methods (the filter criterion for comprehensive evaluation was P-value less than 0.05), thus assessing in which cell subgroups the gene sets were enriched. Finally, we extracted the enrichment scores of the gemcitabine resistance gene set and correlated them with LRG expression levels. The statistical significance of the impact of LRG expression levels on the enrichment scores of gemcitabine resistance genes was evaluated using the Wilcoxon Signed Rank Test, with significance set at P<0.05.

### 2.10 Exploring the influence of LRG on the metabolic function of pancreatic cancer cells

We conducted metabolic analysis on pancreatic cancer single-cell sequencing tissues using the scMetabolism package. The metabolic module of this package combined data from public metabolite databases and literature, annotating and quantifying metabolites in single-cell mass spectrometry analysis data to determine the content and types of metabolites in each cell. Later, we aligned the outcomes of metabolite annotation with metabolic pathway databases like KEGG. Employing enrichment analysis techniques, we evaluated the enrichment of metabolic pathways in individual or multiple cells, delving into the biological functions of these pathways and the structure of metabolic networks. In this investigation, we conducted distinct analyses to examine the metabolic variances between primary lesions and liver metastases of pancreatic cancer, as well as the metabolic distinctions between cells exhibiting high and low LRG expression. The intersection of these analyses represented the metabolic changes mediated by LRG in the process of liver metastasis.

### 2.11 Cell culture

We utilized the human pancreatic cancer cell lines PANC-1 and Bx-PC-3 (Pricella) for our investigation. Cell cultures were maintained in 1640 medium (Pricella) supplemented with 10% fetal bovine serum (Biological Industries, BI) and 1% penicillin-streptomycin (Hyclone). The cells were incubated in a humidified chamber at 37°C with 5% CO2.

### 2.12 siRNA transfection

siRNAs were transfected into both cell lines using riboFECT™ CP Reagent (RIBOBIO). siRNAs were obtained from RIBOBIO. After pre-experiments, it was determined that a concentration of 100nM and the 24-hour incubation for siRNA transfection yielded optimal results.

### 2.13 RNA extraction and qPCR detection

We utilized the RNA easy Isolation Reagent (Vazyme) to extract total RNA from PANC-1 and Bx-PC-3 cells, and employed the HiScript III RT SuperMix for qPCR (+gDNA wiper) (Vazyme) for reverse transcription on a PCR machine to obtain cDNA. The reverse transcription process was as follows: 2ul of RNA was added at 42°C for a reaction time of 2 minutes, along with 4ul of 4x gDNA wiper mix and 10ul of RNase-free ddH2O to remove genomic DNA. Subsequently, 4ul of 5x Hiscript III qRT Supermix was added and incubated at 37°C for 15 minutes, then at 85°C for 5 seconds. Finally, the ChamQ Universal SYBR qPCR Master Mix reagent (Vazyme) was used for fluorescent quantitative PCR. The experiment used a 10ul system: 0.4ul of Forward and Reverse primers each; 3.2ul of ddH2O, 1ul of cDNA, and 5ul of SYBR qPCR Master Mix. The specific reaction steps were as follows: Stage 1: Pre-denaturation: 95°C for 30 seconds. Stage 2: Cycle reaction: 95°C for 10 seconds to 60°C for 30 seconds, for 40 cycles. Stage 3: Melting curve: 95°C for 15 seconds, 60°C for 60 seconds, 95°C for 15 seconds. PAK2 primers, TGFβ-related gene primers, and EMT-related gene primers were designed by us using Oligo7, and along with the internal reference GAPDH primers, were provided by Shanghai Shenggong Company. The PAK2 primers were as follows: F: 5’ CTCCTCCCGTTATTGCC 3’; R: 5’ TGCACGTTTCTGTTACCAC 3’. For additional primer information, refer to **Table 1**. The results of the above qPCR were analyzed using GAPDH as an internal reference, and the fold changes were calculated using the ΔΔCT method (7).

**Table 1:** All the online websites used in this study.

GAPDH:	
F: 5' CAGGAGGCATTGCTGATGAT 3'	R: 5' GAAGGCTGGGGCTCATTT 3'
TGFB1	
F: 5' TACCTGAACCCGTGTTGCTCT 3'	R: 5' CTGCCGCACAACTCCGGTGA 3'
SMAD7	
F: 5' CTCCATCAAGGCTTTCGACT 3'	R: 5' GCTGCATAAACTCGTGGTCA 3'
SNAI1	
F: 5' CCTCACCGGCTCCTTCGTC 3'	R: 5' ACCCAGGCTGAGGTATTCCTT 3'
CDH1	
F: 5' GGTATCTTCCCCGCCCTG 3'	R: 5' CTTCATAGTCAAACACGAGCAG 3'
VIM	
F: 5' AAATGGCTCGTCACCTTCGT 3'	R: 5' AGGGCCATCTTAACATTGAGCA 3'
CDH2	
F: 5' GAGTTTACTGCCATGACGTT 3'	R: 5' GGTTGATCCTTATCGGTCAC 3'

### 2.14 Cell phenotypic experiments

Cell proliferation levels were assessed by adding CCK8 and measuring absorbance at 450nm. CCK8 kit reagents were obtained from GLPBIO company. Control and siRNA-transfected groups were seeded at 3000 cells/well in a 96-well plate, with 100 μl complete medium and 10 μl CCK8 kit added per well. Detection time points were set at 24h, 48h, and 72h using three 96-well plates. After adding CCK8 kit, cells were uniformly incubated for 2 hours, and OD values were measured at 450nm using elx800 Epoch microplate reader.

To assess cell migration ability, scratch assays were conducted. After overnight confluence in a 6-well plate, both cell types were transfected with siRNA. The medium was then replaced with serum-free medium, and scratches were made. Cell migration was recorded at 0h and 24h. Image J software was employed to calculate the migration area and migration rate.

### 2.15 Flow cytometric analysis of cell apoptosis

Confirming the logarithmic growth phase of pancreatic cancer cell lines, cells were then seeded into a 6-well plate with 60,000 cells per well. Based on whether PAK2 in the cell lines was silenced, they were divided into a control group (wild-type) and a knockdown group (PAK2 silenced). After 48 hours, cells from both groups were aspirated into centrifuge tubes, washed twice with cold PBS by centrifugation at 1200 rpm in a low-speed centrifuge, and then resuspended in 1× Binding Buffer at a concentration of 1.0×106/ml. A 100 μl solution was aspirated into the centrifuge tube and 5 μl of PE Annexin V and 5 μl of 7-AAD were added. Cells were vortexed and incubated for 15 minutes at room temperature in the dark. After adding 400 μl of 1× Binding Buffer to each tube, the apoptotic rate of cells was analyzed using a flow cytometer.

### 2.16 Statistical tests

All data analyses in this study were conducted using R (version 4.1.3). Statistical tests were rigorously applied according to their scope. In differential analysis, the t-test method was employed, and the t-statistic was adjusted using empirical Bayesian methods. Correlation analysis in hdWGCNA utilized the Spearman method. Differential comparisons among multiple groups were based on the Wilcoxon Signed Rank Test. The analysis of Real-time Fluorescence Quantitative PCR results for cell lines was conducted using one-way ANOVA. For the results analysis of CCK-8 cell proliferation experiments, two-way ANOVA was applied. The comparison of migration areas after cell scratch experiments was based on Welch’s test. The specific application of statistical testing methods has been described in the corresponding methodology sections. In this study, all tests were considered statistically significant when P-value < 0.05. All the online websites used in this study are visible in **Table 2**.

**Table 2:** Primer sequences.

Database	website
GEO	https://www.ncbi.nlm.nih.gov/geo/
TCGA	https://www.cancer.gov/about-nci/organization/ccg/research/structural-genomics/tcga
CellMarker	http://xteam.xbio.top/CellMarker/
BMC Genome Biology	https://genomebiology.biomedcentral.com/
The human protein atlas	https://www.proteinatlas.org/
Cancer Therapeutics Response Portal	http://portals.broadinstitute.org/ctrp/
Genomics of Drug Sensitivity in Cancer	https://www.cancerrxgene.org/
Ensemble	http://asia.ensembl.org/index.html

## 3 Results

### 3.1 Single-cell sequencing revealed the cellular composition of the pancreatic cancer microenvironment

**Figure 1:**
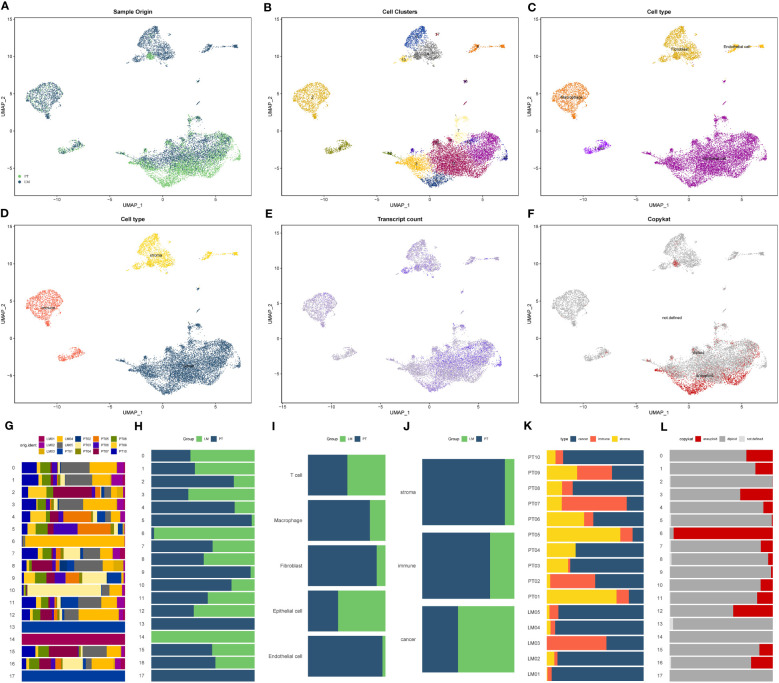
Single-cell composition and expression characteristics of the pancreatic cancer liver metastasis microenvironment. **(A)** Distribution of cells in the pancreatic cancer liver metastasis dataset after UMAP dimensionality reduction and cell cycle correction. PT: Pancreatic cancer primary lesion tissue, LM: Pancreatic cancer liver metastasis tissue. **(B)** Seurat matrix showing the 18 cell subtypes (Cluster 0-17) in liver metastasis tissue. **(C)** Classification of 18 cell subtypes into 5 cell types through a combination of machine learning algorithms and manual annotation: Epithelial cells (Cluster 0, 1, 3, 6, 7, 10, 11, 12, 14, 15, 16), Macrophages (Cluster 2), Fibroblasts (Cluster 4, 5, 13, 17), T cells (Cluster 8), Endothelial cells (Cluster 9). **(D)** Classification of the pancreatic cancer liver metastasis microenvironment into three main components: stroma (fibroblasts, endothelial cells), epithelium (epithelial cells), and immune (T cells, macrophages) based on cell types. **(E)** Gene expression levels of individual cells, with darker colors indicating higher gene expression. **(F)** Copykat algorithm inference results for malignant cells, with red cell subtypes defined as malignant cells. **(G-L)** Proportions of various cell subtypes and cell types in different groups. **(G)** Proportions of 18 cell subtypes in 15 samples. **(H)** Proportions of 18 cell subtypes in pancreatic cancer primary lesion and liver metastasis. **(I)** Proportions of 5 annotated cell types in pancreatic cancer primary lesion and liver metastasis. **(J)** Proportions of the three main TME components in pancreatic cancer primary lesion and liver metastasis. **(K)** Proportions of the three main TME components in 15 samples. **(L)** Proportions of cells identified as malignant by the copykat algorithm in 15 samples.

The flowchart of this study was presented in **Supplementary Figure S1**. After the integration and processing of single-cell sequencing data from pancreatic cancer, we obtained the expression matrix for the GSE154778 pancreatic cancer liver metastasis dataset. The original matrix included 15,707 cells and 23,754 genes. After secondary filtering, we selected 13,922 cells and 23,754 genes for further analysis. Through UMAP dimensional reduction, we categorized all cells into 15 subgroups (Cluster 0-14). However, cell cycle analysis using Seurat’s built-in dataset suggested a significant impact of the cell cycle on the dimensional reduction results. Hence, we accounted for the cell cycle. After this correction, the distribution of cells in the primary and metastatic lesions exhibited a more uniform pattern (**Figure 1A**). The Seurat matrix contained 18 cell subgroups (Cluster 0-17) (**Figure 1B**). Through the identification by machine learning algorithms and manual annotation, we determined that these 18 cell subgroups originated from five cell types: epithelial cells (Cluster 0, 1, 3, 6, 7, 10, 11, 12, 14, 15, 16), macrophages (Cluster 2), fibroblasts (Cluster 4, 5, 13, 17), T cells (Cluster 8), and vascular endothelial cells (Cluster 9) (**Figure 1C**). Based on the attribution to cell types, we further divided the pancreatic cancer tumor microenvironment into stroma (fibroblasts, vascular endothelial cells), epithelium (epithelial cells), and immune (T cells, macrophages) as the three main components (**Figure 1D**). All cells showed relatively high gene expression, with epithelial cells being the most significant (**Figure 1E**). Finally, the Copy Number Karyotyping of Aneuploid Tumors (CopyKAT) algorithm defined epithelial cells as non-diploid cells, namely malignant cells. Therefore, all malignant cells in pancreatic cancer tissue originated from epithelial cells (**Figure 1F**). **Figures 1G-L** showed the proportions of various cell subgroups and cell types in different groups.

### 3.2 hdWGCNA identified key modules in the pancreatic cancer liver metastasis process

**Figure 2:**
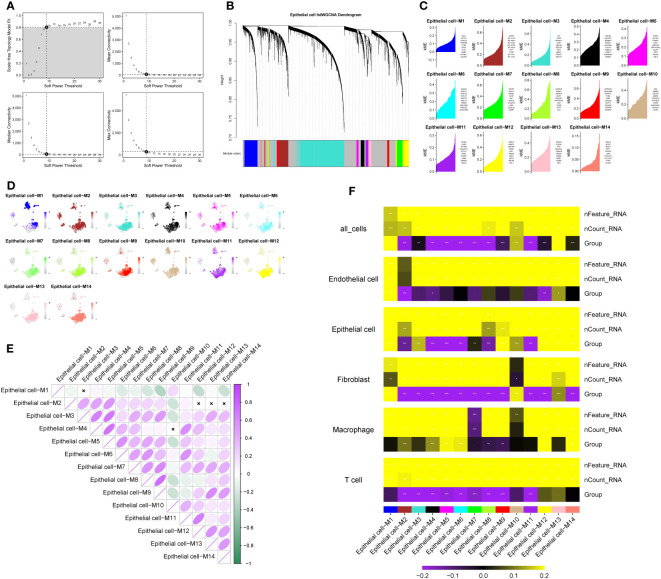
Identification of key modules in pancreatic cancer liver metastasis based on hdWGCNA. **(A)** Soft-threshold selection process, 9 is the optimal soft-threshold for constructing a co-expression network with a scale-free network structure. **(B)** Dendrogram visualizing the hierarchical structure of the co-expression network, where each module color represents a distinct module. **(C)** Fourteen modules highly correlated with pancreatic cancer liver metastasis and their central genes. **(D)** Enrichment of module genes at the single-cell level, with darker colors indicating high enrichment of module genes in specific cell subtypes. **(E)** Inter-module correlation analysis, showing the strength of correlation between each module and all other modules. Purple indicates positive correlation, green indicates negative correlation. Darker colors represent stronger correlation, while white indicates no significant association between two modules. **(F)** Heatmap illustrating the correlation between modules and pancreatic cancer liver metastasis. Colors represent the strength of correlation, with yellow indicating positive correlation and purple indicating negative correlation. The intensity of the color reflects the strength of the correlation. represents P<0.1, * represents P<0.05, ** represents P<0.01, *** represents P<0.001.

Using the TestSoftPowers function in the hdWGCNA package, we tested different soft power values to find a suitable value that gives the constructed co-expression network a scale-free network structure. Ultimately, 9 was selected as the optimal soft threshold, making the network’s topology most consistent with the actual biological relationships (**Figure 2A**). Subsequently, we explored the relationships between gene modules in the co-expression network by measuring gene expression similarity, calculating the topological overlap matrix, and performing hierarchical clustering analysis. The hierarchical structure of the co-expression network was visualized using a dendrogram (**Figure 2B**). Next, we obtained genes with representative expression patterns in each module (module eigengenes) and calculated the correlation of each gene with the module eigenvector (module connectivity, kME). We visualized the relationships between genes within modules using a connectivity plot (**Figure 2C**). Next, we pinpointed the top 25 central genes, namely, genes with the highest connectivity, and illustrated the central gene signature scores for each module, based on their normalized expression (**Figure 2D**). Furthermore, correlation analysis demonstrated the strength of correlations between each module and all other modules (**Figure 2E**). Finally, a correlation heatmap displayed the strength of the correlation between each module and pancreatic cancer liver metastasis. We selected modules 13 (R=0.68, P<0.05), 6 (R=-0.43, P<0.05), and 14 (R=0.3, P<0.05) as meeting the criteria of module correlation coefficient > 0.3 and P-value < 0.05 (**Figure 2F**). All modules comprised a total of 294 genes, with module 13 having 175 genes, module 6 having 57 genes, and module 14 having 62 genes. Differential analysis revealed 1,780 genes with significant differential expression between epithelial cells in pancreatic cancer metastases and primary lesions, meeting the criterion of P < 0.05. The intersection of 139 genes between the two sets was recognized as the final set of pancreatic cancer LRG identified from single-cell sequencing data.

### 3.3 COX regression model combined with machine learning algorithms identified PAK2 as a pancreatic cancer liver metastasis-related gene

**Figure 3:**
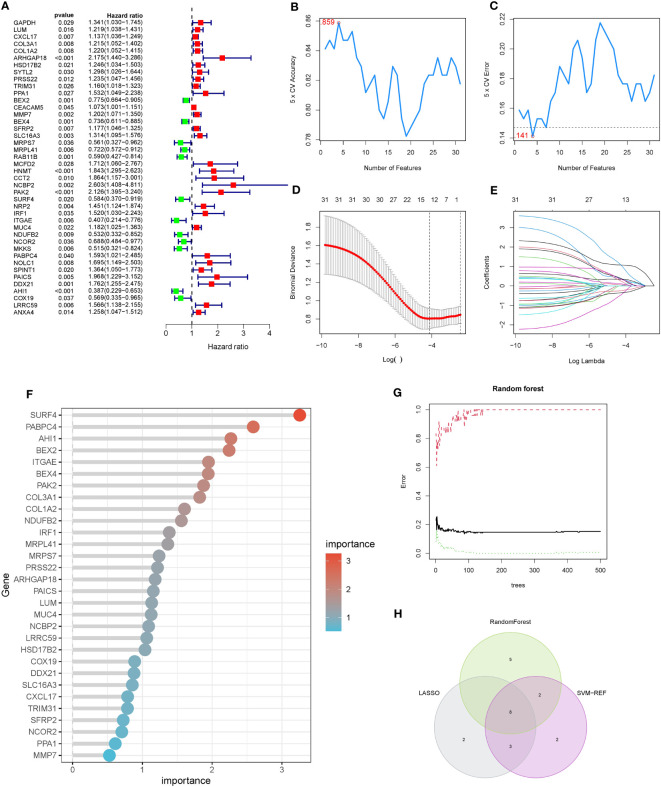
COX regression model combined with machine learning algorithms for the selection of pancreatic cancer liver metastasis-related genes. **(A)** Results of single-factor COX regression analysis on 139 genes in the TCGA cohort. Hazard ratio values less than 1 indicate protective factors for the prognosis of pancreatic cancer patients, while values greater than 1 indicate risk factors. **(B, C)** SVM-RFE machine learning feature selection. The top 15 genes were extracted based on the average ranking from ten-fold cross-validation, including COL1A2, PRSS22, PAK2, SURF4, IRF1, PABPC4, AHI1, and ANXA4. **(D, E)** LASSO regression model feature selection. Thirteen genes were extracted based on the selection of lambda.min, including COL1A2, PRSS22, PAK2, SURF4, IRF1, PABPC4, AHI1, and ANXA4. **(F, G)** Feature selection using random forest analysis. The top 15 genes were extracted based on the ranking of gene importance, including COL1A2, PRSS22, PAK2, SURF4, IRF1, PABPC4, AHI1, and ANXA4. **(H)** Intersection of the gene sets obtained from the three machine learning methods, resulting in a final set of 8 genes.

To mitigate the risk of false positives, we employed three machine learning methods to further refine the selection of pancreatic cancer LRG identified through single-cell analysis. Considering the significant impact of liver metastasis on the prognosis of pancreatic cancer patients, we initiated univariate COX regression analysis on the gene set within the TCGA cohort. The outcomes revealed that out of the 139 genes, 42 were linked to the prognosis of pancreatic cancer patients, comprising 30 risk factors and 12 protective factors (**Figure 3A**). Next, we evaluated the diagnostic performance of the selected 42 genes using a univariate logistic regression model in the GSE71729 cohort. Thirty-one genes showed good discriminative ability between primary lesions and liver metastases in pancreatic cancer (**Table 3**). Ultimately, we performed feature selection on the 31 genes using three machine learning algorithms. SVM-RFE, employing ten-fold cross-validation, identified the top 15 genes based on the average ranking across 10 folds (**Figures 3B, C**). In LASSO regression, we determined lambda.min and identified 13 genes screened by the LASSO model (**Figures 3D, E**). Following random forest analysis (8), we extracted the top 15 genes based on gene importance ranking (**Figures 3F, G**). Eight genes were common in the results of the three machine learning methods: COL1A2, PRSS22, PAK2, SURF4, IRF1, PABPC4, AHI1, ANXA4 (**Figure 3H**). Among these genes, six genes had contradictory prognosis and expression patterns, so they were excluded. IRF1 performed poorly in the TCGA cohort, while PAK2 consistently showed excellent performance in all analyses. Hence, PAK2 was chosen as the pivotal gene in the progression of pancreatic cancer liver metastasis for subsequent investigations.

**Table 3:** Univariate logistic regression model evaluation results.

Gene ID	P-value
LUM	9.00E-08
CXCL17	0.000932
COL3A1	4.02E-06
COL1A2	5.55E-06
ARHGAP18	0.002463
HSD17B2	0.035921
PRSS22	0.004958
TRIM31	0.00151
PPA1	0.004154
BEX2	2.74E-06
MMP7	5.77E-06
BEX4	1.96E-07
SFRP2	6.94E-08
SLC16A3	0.001743
MRPS7	0.005893
MRPL41	2.32E-07
NCBP2	0.010999
PAK2	0.000298
SURF4	8.55E-05
IRF1	0.000876
ITGAE	0.08793
MUC4	0.007265
NDUFB2	0.048649
NCOR2	0.013449
PABPC4	0.006308
PAICS	0.000178
DDX21	0.043241
AHI1	0.063661
COX19	8.80E-07
LRRC59	0.09702
ANXA4	4.48E-05

### 3.4 Various lines of evidence consistently underlined the substantial role of PAK2 in the progression of pancreatic cancer liver metastasis

**Figure 4:**
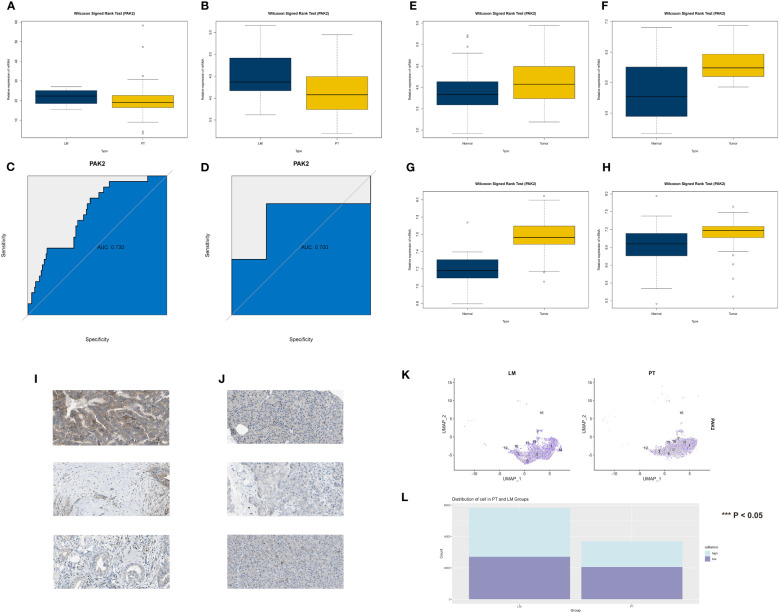
Multiple lines of evidence reconfirming the significant role of PAK2 in the process of pancreatic cancer liver metastasis. **(A-D)** Validation of the role played by PAK2 in the process of pancreatic cancer liver metastasis. **(A)** In the TCGA cohort, the gene expression of PAK2 in patients with hematogenous metastasis (M1 stage) is significantly higher than in patients without hematogenous metastasis (M0 stage). **(B)** In the GSE71729 cohort, the gene expression of PAK2 in patients with pancreatic cancer liver metastasis is significantly higher than in patients without liver metastasis. **(C, D)** ROC analysis showing the area under the curve (AUC) in the GSE19279 cohort (AUC = 0.73) and GSE71729 cohort (AUC = 0.7). **(E-H)** Validation of the role played by PAK2 in the occurrence of pancreatic cancer. Expression trends and rank sum test results of PAK2 in the GSE15471, GSE62165, GSE62452, and GSE71729 cohorts. **(I, J)** Protein-level evidence of PAK2 mediating the occurrence of pancreatic cancer provided by the HPA database. Left image: Normal pancreatic tissue (low PAK2 expression), right image: Pancreatic cancer tissue (high PAK2 expression). **(K)** Comparative analysis in single-cell sequencing of pancreatic cancer cells reveals that the expression level of PAK2 in pancreatic cancer cells in liver metastatic tissues is significantly higher than in cells from the primary site. **(L)** High expression of PAK2 in pancreatic cancer cells compared to low expression of PAK2 in pancreatic cancer cells in the primary tumor and liver metastatic lesions of pancreatic cancer. The p-value represents the statistical test results for the difference in quantity.

We confirmed the significance of PAK2 across multiple datasets and databases, investigating its implications in the initiation and advancement of pancreatic cancer. In the TCGA cohort, patients with hematogenous metastasis (M1 stage) exhibited significantly higher expression of the PAK2 gene compared to patients without hematogenous metastasis (M0 stage) (**Figure 4A**). Moreover, examination of the GSE71729 dataset indicated a notable elevation in PAK2 gene expression levels among patients with pancreatic cancer and liver metastasis, as opposed to those without liver metastasis (**Figure 4B**). ROC analysis indicated good discriminative ability of PAK2 for pancreatic cancer liver metastasis in the GSE19279 cohort (AUC=0.73, **Figure 4C**) and GSE71729 cohort (AUC=0.7, **Figure 4D**). Additionally, we assessed the oncogenic role of PAK2. In the GSE15471 (**Figure 4E**), GSE62165 (**Figure 4F**), GSE62452 (**Figure 4G**), and GSE71729 (**Figure 4H**) cohorts, the Wilcoxon test showed statistically significant differences in PAK2 expression between pancreatic cancer and normal individuals, with elevated expression of PAK2 in pancreatic cancer patients. Evidence at the protein level, indicating PAK2’s role in mediating pancreatic cancer, was provided by the HPA database (**Figures 4I, J**). Finally, in single-cell sequencing of pancreatic cancer cells, a comparison of PAK2 expression revealed a significantly higher level of PAK2 in pancreatic cancer cells from liver metastatic tissues compared to primary pancreatic cancer tissues (**Figures 4K, L**).

### 3.5 GSVA analysis reveals PAK2 promoted liver metastasis from the primary site through the TGF-beta signaling pathway

**Figure 5:**
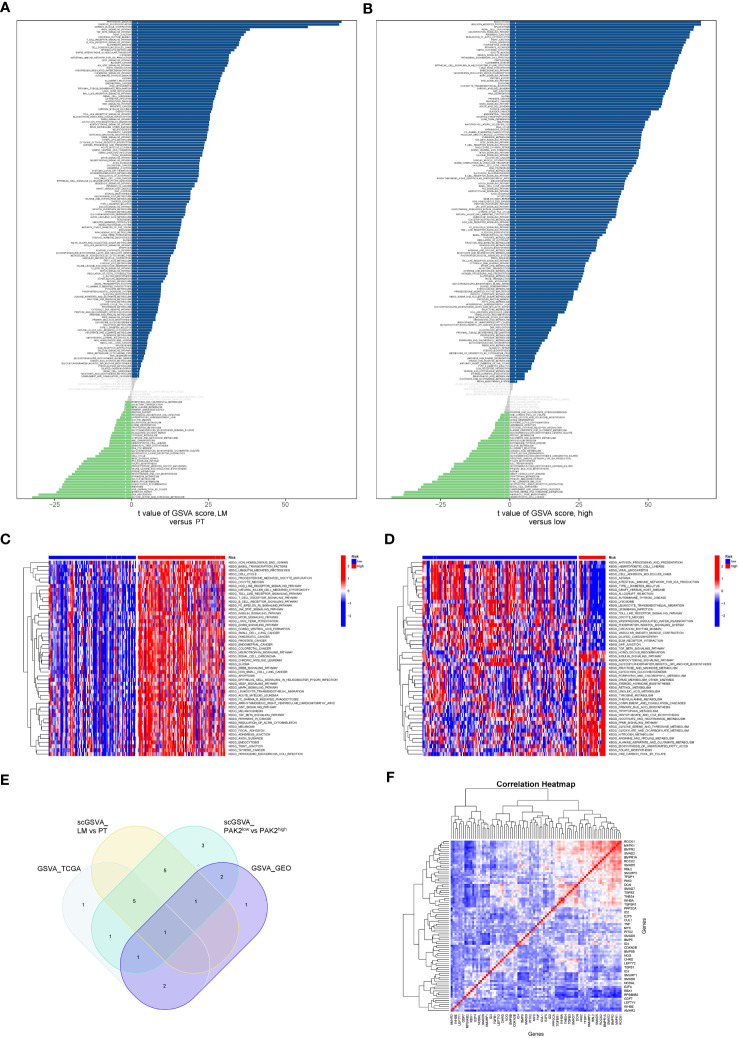
GSVA analysis reveals signaling pathways regulated by PAK2. **(A)** Differential activation of signaling pathways and gene functions in pancreatic cancer cells from primary tumors and liver metastases in the GSE154778 dataset. **(B)** Differential activation of signaling pathways and gene functions between pancreatic cancer cells with high PAK2 expression and those with low PAK2 expression in the GSE154778 dataset. **(C)** GSVA analysis results in pancreatic cancer tissues from the TCGA cohort, divided into low and high PAK2 expression groups based on the median PAK2 expression. **(D)** GSVA analysis results for the primary tumor and metastatic tumor groups in the GSE71729 cohort. **(E)** Significantly upregulated signaling pathways in all four GSVA analyses, with a focus on the TGF-Beta signaling pathway. “scGSVA_LM vs PT” represents the comparison between the primary tumor and liver metastasis in pancreatic cancer; “scGSVA_PAK2^low^ vs PAK2^high^” represents the comparison between pancreatic cancer cells with low PAK2 expression and those with high PAK2 expression; “GSVA_TCGA” indicates the comparison between samples with high PAK2 expression and low PAK2 expression in the TCGA cohort; “GSVA_GEO” signifies the comparison between samples with high PAK2 expression and low PAK2 expression in the GEO cohort. **(F)** Correlation analysis showing significant associations between various genes in the TGF-Beta signaling pathway and PAK2. Red represents high expression, and blue represents low expression.

We conducted single-cell GSVA on pancreatic cancer cells from the primary site and liver metastatic site in the GSE154778 dataset. The results demonstrated enhanced activation of multiple signaling pathways in cancer cells from the liver metastatic site (**Figure 5A**). Furthermore, to obtain a sufficient number of pancreatic cancer cells, we integrated two datasets, GSE155698 and GSE154778, extracted cancer cells, and divided them into low and high PAK2 expression groups. Following GSVA analysis on both cell types, we identified abnormal activation of numerous signaling pathways in PAK2-high expressing cells (**Figure 5B**). Additionally, GSVA analysis was performed on bulk sequencing data. We categorized pancreatic cancer tissues from the TCGA cohort into low and high PAK2 expression groups according to the median PAK2 expression. Subsequently, we performed GSVA analysis on both groups (**Figure 5C**). GSVA analysis was also performed on the primary and metastatic groups in the GSE71729 cohort (**Figure 5D**). Finally, the TGF-Beta signaling pathway was consistently upregulated in all four GSVA analyses, indicating it as the key pathway primarily regulated by PAK2 in mediating pancreatic cancer liver metastasis (**Figure 5E**). Further correlation analysis revealed significant associations between various genes in the TGF-Beta signaling pathway and PAK2 (**Figure 5F**). Therefore, we identify the TGF-Beta signaling pathway as the key signaling pathway regulated by PAK2 in mediating pancreatic cancer liver metastasis. In addition to signaling pathways, we observed that the elevation of PAK2 was correlated with increased malignant behaviors of pancreatic cancer, such as angiogenesis (logFC=0.011, P<0.05), and epithelial-mesenchymal transition (logFC=0.01, P<0.05).

### 3.6 PAK2-mediated differentiation alterations contributed to the malignant behavior of PDAC

**Figure 6:**
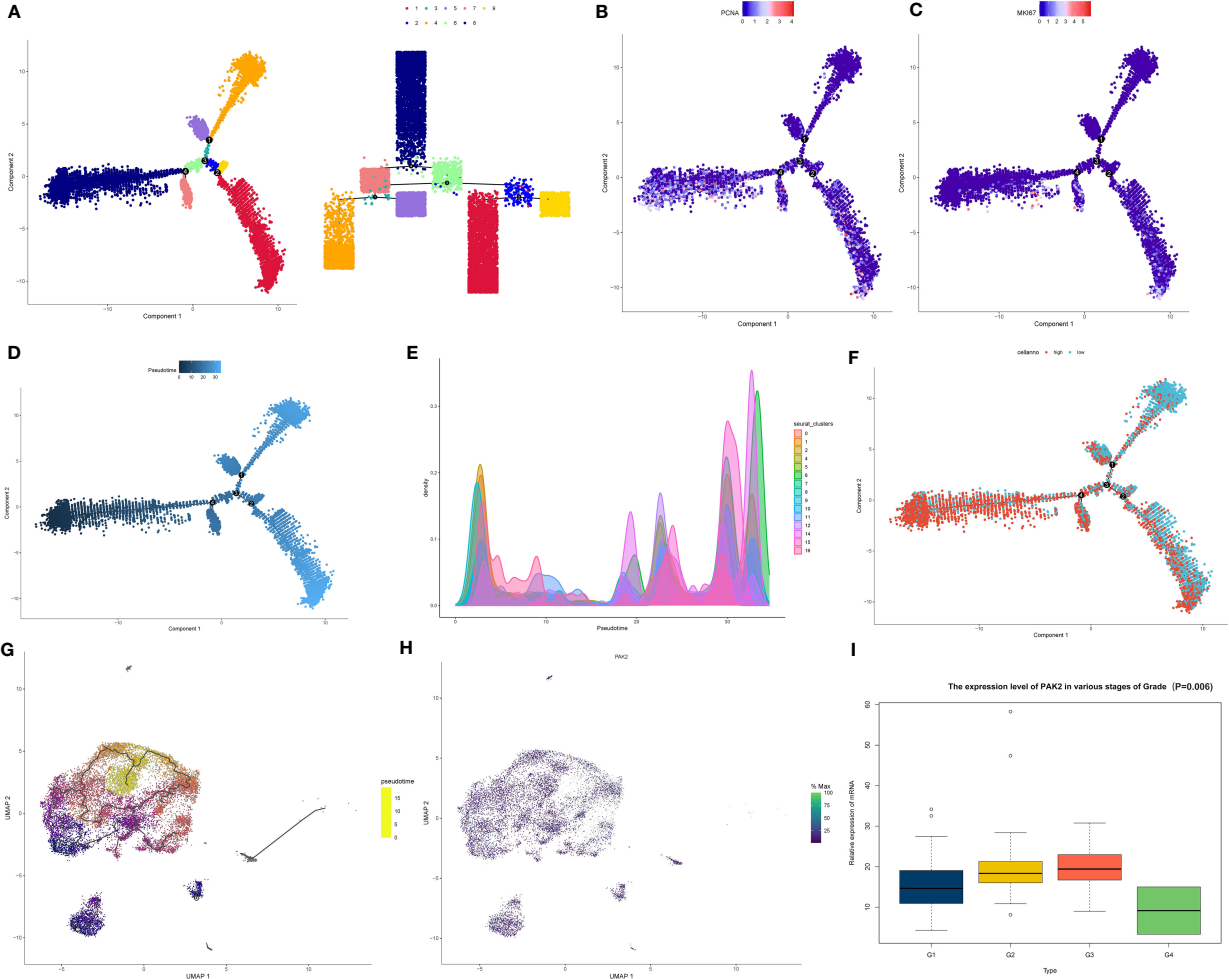
PAK2 impact on pancreatic cancer cell differentiation revealed by pseudotime analysis. **(A)** Pseudotime analysis based on the monocle2 package reveals four key branching points and nine branches in pancreatic cancer tissue. **(B, C)** Expression of cell cycle genes (PCNA, MKI67) at various branches. **(D)** After determining branch 8 as the starting point of the developmental trajectory, pseudotime increases along the direction of the branch (from dark to light), reflecting the gradual maturation of cell differentiation levels. **(E)** Developmental status of each branch cell and pancreatic cancer subtype. **(F)** Distribution of pancreatic cancer cells with high PAK2 expression and low PAK2 expression on the pseudotime trajectory. **(G)** Pseudotime analysis based on the monocle3 package showing the differentiation trajectory of pancreatic cancer cell subtypes. **(H)** Expression of PAK2 in various subtypes. **(I)** Association between the expression levels of PAK2 in TCGA samples and the “Grade” classification of pancreatic cancer.

In this investigation, we explored the influence of PAK2 on the differentiation and development of pancreatic cancer cells. Firstly, utilizing the monocle2 package, we performed developmental inference and trajectory analysis on pancreatic cancer cells to gain insights into their developmental trajectory and potential cell fate decisions. Pseudotime analysis revealed four critical branch points in pancreatic cancer tissue, representing pivotal turning points in the developmental process of cancer cells. Additionally, we observed nine distinct branches, reflecting potential developmental trajectories of cancer cells at these critical time points (**Figure 6A**). These branches showcased the heterogeneity and complexity of cancer cells within pancreatic cancer tissue. To determine the starting point for developmental trajectories, we assessed the expression of cell cycle genes (PCNA, MKI67). Branches displaying elevated expression of cell cycle genes were often associated with a more primitive state along the developmental trajectory (**Figures 6B, C**). Notably, the cell subset in branch 8 was identified as being in the most primitive stage of pancreatic cancer cell development and was designated as the starting point for the trajectory, allowing us to assess the differentiation status of cells and subgroups within pancreatic cancer (**Figures 6D, E**). PAK2-high expressing cells showed a stronger tendency to be in the early branches of the developmental trajectory, while PAK2-low expressing cells, comparatively, exhibited a more mature state of differentiation (**Figure 6F**). According to the results of the Beam algorithm, PAK2 underwent significant changes during the pseudotime process (P<0.05). Thus, we preliminary concluded that PAK2 leads to a less differentiated state in pancreatic cancer cells. Subsequently, using the latest monocle3 package, we conducted pseudotime analysis on pancreatic cancer cells once again. After determining the differentiation starting point using the same method, we found compelling evidence confirming that PAK2 promoted the low differentiation of pancreatic cancer cells (**Figures 6G, H**). Finally, we performed clinical data correlation analysis in the TCGA cohort, and the low differentiation of pancreatic cancer directly manifested in the clinical data’s “Grade” classification. Based on a logistic regression model, we identified a significant association between PAK2 and Grade (P=0.006), with elevated PAK2 levels leading to higher Grade classifications (**Figure 6I**).

### 3.7 Cellular communication highlighted the significant impact of PAK2 on the interaction between ductal cells and tumor microenvironment cells

**Figure 7:**
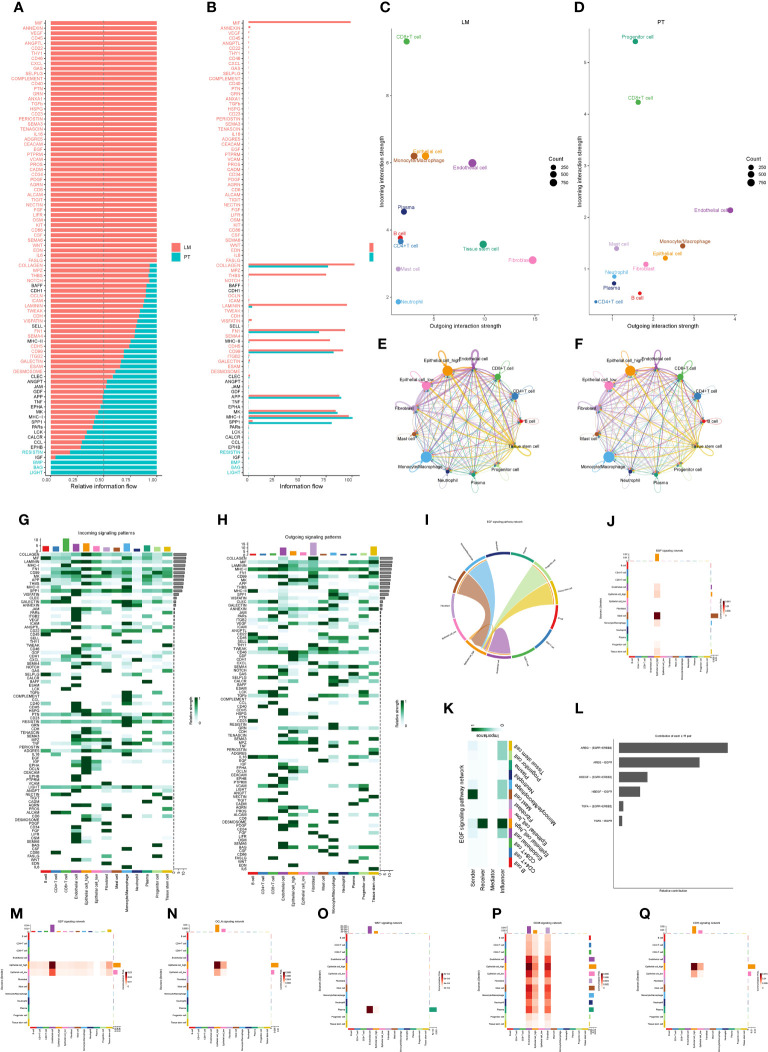
Cell communication network reveals significant role of high PAK2 expression cancer cells in liver metastasis. **(A, B)** Cell communication networks in pancreatic cancer primary and liver metastasis show significant differences in signaling communication. **(C, D)** Pancreatic cancer cells exhibit similar outgoing communication in primary and liver metastasis, while the incoming communication shows a larger difference in liver metastasis. **(E, F)** Interaction quantity and interaction weight/strength of various cells in the pancreatic cancer tumor microenvironment (TME) in the communication network. **(G, H)** Contribution of various cells in the pancreatic cancer TME as signal senders and receivers to various signaling pathways. **(I-L)** Specific analysis results of the EGF signaling pathway. **(I)** Sine wave graph of cell communication in the EGF signaling pathway. **(J, K)** Contribution heatmap of each cell communication in the EGF signaling pathway. **(L)** Receptor with the most frequent communication in the EGF signaling network. **(M-Q)** Contribution heatmap of cell communication in various signaling pathways, including GDF, OCLN, WNT, CD46, and CDH.

Applying the CellChat package, we compared the cell communication networks between primary tumors and liver metastases in pancreatic cancer, uncovering significant differences in signaling communication. We observed that, compared to primary tumors in the pancreas, the signaling communication network in liver metastases exhibited significant activation, including pathways such as MIF, VEGF, and CD45. Results from the cell communication analysis indicated a more complex interaction among cells in liver metastases, suggesting a potential critical role in the metastatic process (**Figures 7A, B**). The outdegree (outgoing) of pancreatic cancer cells remained relatively consistent between primary and liver metastatic sites, while the indegree (incoming) showed a considerable difference. This suggested that cancer cells in liver metastases experienced more external regulation or signal input from other cells (**Figures 7C, D**). Next, we concentrated on analyzing the distinctions in the function of cancer cells with high PAK2 expression compared to those with low PAK2 expression in the signaling communication network. The number and strength of interactions for high PAK2-expressing cancer cells were significantly higher than those for low PAK2-expressing cancer cells (**Figures 7E, F**). Moreover, in shared signaling communication networks involving both cell types (e.g., COLLAGEN, LAMININ, FN1, MK, and THBS), the contribution of high PAK2-expressing cancer cells was markedly greater than that of low PAK2-expressing cancer cells. Some signaling communication networks (CD46, HSPG, EGF, CEACAM, PDGF, and EDN) exclusively involved high PAK2-expressing cancer cells, with no participation from low PAK2-expressing cancer cells (**Figures 7G, H**). We specifically presented the analysis results for the EGF signaling pathway, where high PAK2-expressing cancer cells might play a crucial role as key signal transduction nodes, particularly as signal receivers. This interaction leaded to reciprocal communication with monocytes/macrophages, mast cells, progenitor cells, and tissue stem cells, while low PAK2-expressing cancer cells had minimal involvement in this signaling pathway (**Figures 7I-K**). Within this network, AREG-(EGFR+ERBB2) receptor communication was particularly close (**Figure 7L**). Finally, we also illustrated the performance of both cell types in signaling pathways such as GDF, OCLN, WNT, CD46, and CDH (**Figures 7M-Q**).

### 3.8 PAK2 regulated the activity of various transcription factors leading to complex tumor behaviors

**Figure 8:**
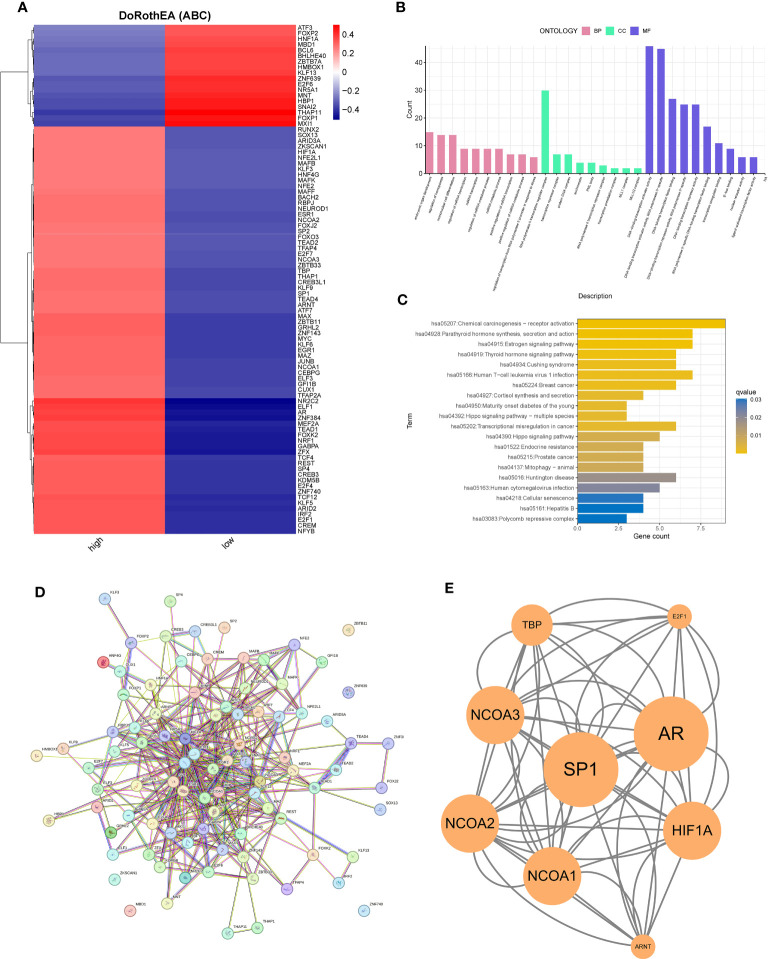
PAK2-regulated transcription factor network and functional enrichment analysis. **(A)** Prediction of transcription factor activity between two cell types based on PAK2 expression, showing transcription factors with significantly different activity. **(B)** GO enrichment analysis of transcription factors, highlighting significant enrichment in molecular function, cellular component, and other biological pathways. **(C)** KEGG enrichment analysis revealing the involvement of PAK2-regulated transcription factors in multiple pathways and functional enrichments. **(D)** Protein-protein interaction (PPI) network of transcription factors showing a complex interaction network. **(E)** Extraction of a key subnetwork playing a crucial role in the protein-protein interaction network using Cytoscape software.

Using the DoRothEA package, we explored the transcription factors regulated by PAK2. In pancreatic cancer cells, substantial evidence supported the involvement of PAK2 in the regulation of 90 transcription factors, including 72 positive regulators and 18 negative regulators (**Figure 8A**). To decipher the molecular functions and signaling pathways regulated by these transcription factors, we conducted GO enrichment analysis and KEGG enrichment analysis. The results indicated significant enrichment in various biological pathways, including (1) Molecular Function: regulation of miRNA transcription, miRNA metabolic process, etc.; (2) Molecular Function: RNA polymerase II transcription regulator complex, transcription repressor complex, etc.; (3) Cellular Component: DNA-binding transcription activator activity, DNA-binding transcription activator activity, RNA polymerase II-specific, etc. (**Figure 8B**). KEGG analysis revealed enrichment in pathways such as the Estrogen signaling pathway, Thyroid hormone signaling pathway, Hippo signaling pathway, and Cellular senescence (**Figure 8C**). As transcription factors exerted their functions as proteins, we utilized PPI to analyze the network of their interactions at the protein level. The PPI network revealed a complex interaction network among transcription factors (**Figure 8D**). Using the MCODE algorithm, we extracted the core network, identifying SP1, AR, the NCOA family, and HIF1A as key players in the transcription factor network primarily regulated by PAK2 (**Figure 8E**).

### 3.9 High expression of PAK2 promoted gemcitabine resistance in pancreatic cancer

**Figure 9:**
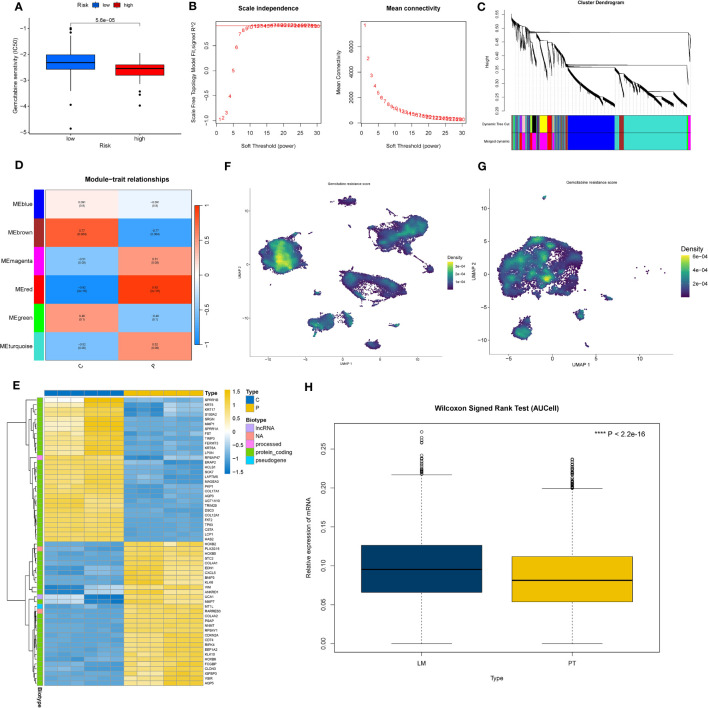
PAK2 and gemcitabine resistance in pancreatic cancer. **(A)** Sensitivity differences to gemcitabine treatment between PAK2 high-expression and low-expression groups in the TCGA pancreatic cancer cohort. **(B)** Construction of a co-expression network based on the GSE140077 dataset using the optimal soft threshold “11.” **(C)** Merging similar modules to obtain six modules related to gemcitabine resistance. **(D)** Selection of modules strongly correlated with gemcitabine resistance (red and brown modules). **(E)** Differential analysis of the two groups in GSE140077, heatmap showing genes significantly upregulated/downregulated in gemcitabine-resistant cell lines. **(F)** Enrichment analysis of gemcitabine resistance genes in pancreatic cancer cells based on single-cell data using the AUCell algorithm. **(G)** Boxplot visualizing the relationship between PAK2 expression and gemcitabine resistance gene enrichment scores.

In the TCGA dataset, we scrutinized the variations in sensitivity to common chemotherapy drugs between the low PAK2 expression group and the high PAK2 expression group. The results showed that increased expression of PAK2 reduces the sensitivity of pancreatic cancer to gemcitabine treatment (**Figure 9A**). To further explore the mechanism by which PAK2 induces gemcitabine resistance, we first identified gemcitabine resistance-related genes in pancreatic cancer based on the GSE140077 dataset. In GSE140077, all cell lines were divided into resistant and non-resistant groups. We constructed a co-expression network with the optimal soft threshold “11” (**Figure 9B**), and after merging the obtained modules, we obtained six modules (**Figure 9C**). We selected modules strongly associated with gemcitabine resistance (R > 0.7, P < 0.05) (red and brown modules) (**Figure 9D**) and genes that were significantly differentially expressed between the two groups (logFC > 1, P < 0.05) (**Figure 9E**). This resulted in 27 gemcitabine resistance-related genes. The enrichment analysis of gemcitabine resistance genes based on the AUCell algorithm in pancreatic cancer single-cell data is shown in the figure. Clearly, gemcitabine resistance genes were significantly enriched in pancreatic cancer cells (**Figures 9F, G**). Among them, pancreatic cancer cells in the cluster 8 with TIMP1+/FN1+ showed the strongest resistance, while the cluster 1 with CLDN18-/CEACAM5- and the cluster 7 with HSPA6+/NEAT1+ showed milder resistance. Finally, we visualized the relationship between PAK2 expression and gemcitabine resistance gene enrichment scores. Cells with high PAK2 expression exhibited higher gemcitabine resistance scores, indicating a more pronounced resistance effect (**Figure 9H**).

### 3.10 High expression of PAK2 leaded to abnormal metabolic changes in cancer cells

**Figure 10:**
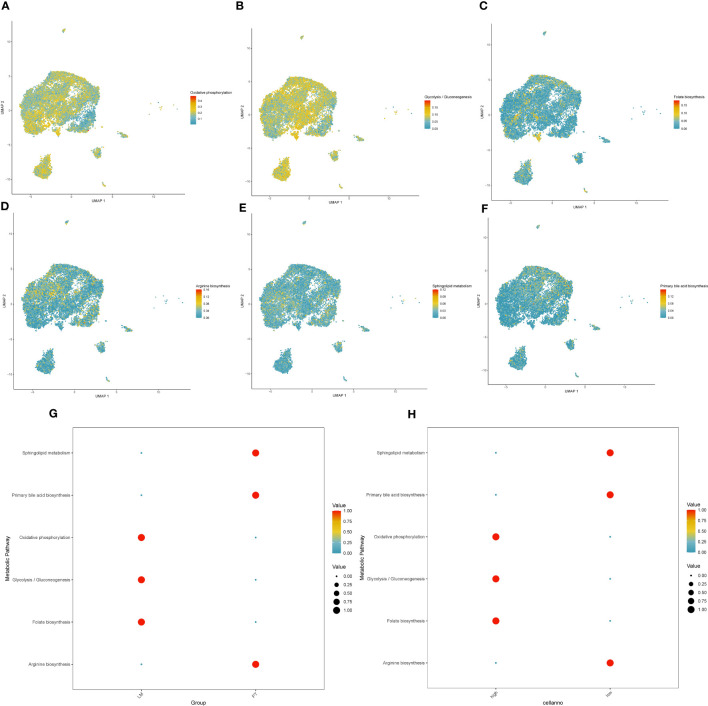
Expression changes in metabolic pathways regulated by PAK2 in pancreatic cancer liver metastasis. **(A-C)** Upregulated metabolic pathways in PAK2 high-expression cells compared to low-expression cells. **(A)** Oxidative phosphorylation. **(B)** Glycolysis/Gluconeogenesis. **(C)** Folate biosynthesis. **(D-F)** Downregulated metabolic pathways in PAK2 high-expression cells compared to low-expression cells. **(D)** Arginine biosynthesis. **(E)** Sphingolipid metabolism. **(F)** Primary bile acid biosynthesis. **(G, H)** Expression patterns of the six significantly altered metabolic pathways in the comparison between primary tumor and liver metastasis and between PAK2 high-expression and low-expression cells. **(G)** Primary tumor vs. liver metastasis. **(H)** PAK2 high-expression cells vs. PAK2 low-expression cells.

There were significant metabolic changes between the liver metastasis and primary lesion of pancreatic cancer. In our analysis, we found 37 metabolic pathways significantly upregulated and 31 metabolic pathways significantly downregulated in the liver metastasis of pancreatic cancer. Applying a similar approach to assess PAK2 high-expression cells and PAK2 low-expression cells, we observed that PAK2 triggered the upregulation of 17 metabolic pathways and the downregulation of 25 metabolic pathways in pancreatic cancer cells. Upon intersection, we identified 23 metabolic pathways implicated in PAK2-promoted pancreatic cancer liver metastasis, comprising 5 upregulated and 18 downregulated metabolic pathways. We selected the most significant three upregulated metabolic pathways and three downregulated metabolic pathways for visualization. The upregulated pathways were Oxidative Phosphorylation (**Figure 10A**), Glycolysis/Gluconeogenesis (**Figure 10B**), and Folate Biosynthesis (**Figure 10C**). The downregulated pathways were Arginine Biosynthesis (**Figure 10D**), Sphingolipid Metabolism (**Figure 10E**), and Primary Bile Acid Biosynthesis (**Figure 10F**). The expression of these six significantly altered metabolic pathways in the primary lesion/liver metastasis (**Figure 10G**) and PAK2 high-expression/low-expression cells (**Figure 10H**) was shown in the figures.

### 3.11 *In vitro* experiments provided evidence that the inhibition of PAK2 results in diminished proliferation and invasion capabilities in pancreatic cancer cell lines

**Figure 11:**
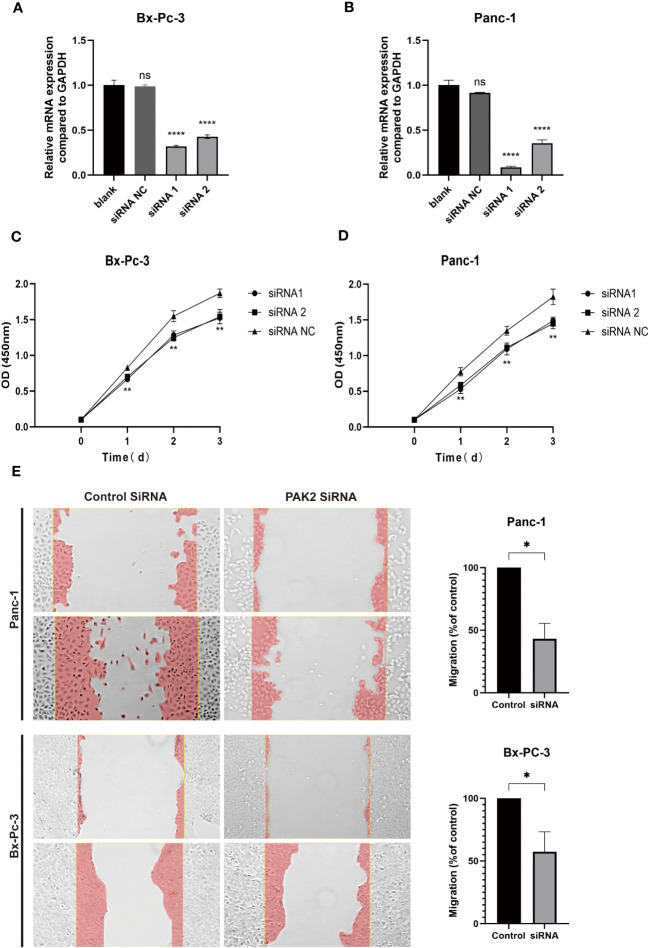
*In vitro* experiments demonstrate that the inhibition of PAK2 leads to reduced proliferation and invasion capabilities in pancreatic cancer cell lines. **(A, B)** Real-time fluorescence quantitative PCR was conducted on PANC-1 and Bx-Pc-3 cells (n=3, one-way ANOVA, P<0.0001). **(C, D)** CCK-8 cell proliferation experiments were performed on PANC-1 and Bx-Pc-3 cells (n=4, two-way ANOVA, P<0.01). **(E)** Comparison of migration areas after scratch assays on PANC-1 and Bx-Pc-3 cells (n=3, Welch’s test, P<0.05). ns represents no statistical significance, represents * represents P<0.05, **** represents P<0.0001.

In **Figure 11**, subsequent to siRNA transfection in pancreatic cancer cell lines PANC-1 and Bx-PC3 (**Figures 11A, B**), qPCR analysis demonstrated a significant reduction in PAK2 expression. Examination of proliferation post-PAK2 knockdown using CCK8 (**Figures 11C, D**) showcased diminished OD values in comparison to the control group, indicative of PAK2 knockdown hindering the proliferation of pancreatic cancer cell lines PANC-1 and Bx-PC-3. The effect of PAK2 knockdown on migration was assessed via scratch assays (**Figure 11E**). As depicted in the figure, the migration area in the experimental group was notably lower than that in the control group, underscoring that PAK2 knockdown hampers the migration of pancreatic cancer cell lines PANC-1 and Bx-PC3.

### 3.12 The results of flow cytometry indicated a significant reduction in the apoptotic levels of pancreatic cancer cells upon downregulation of PAK2

**Figure 12:**
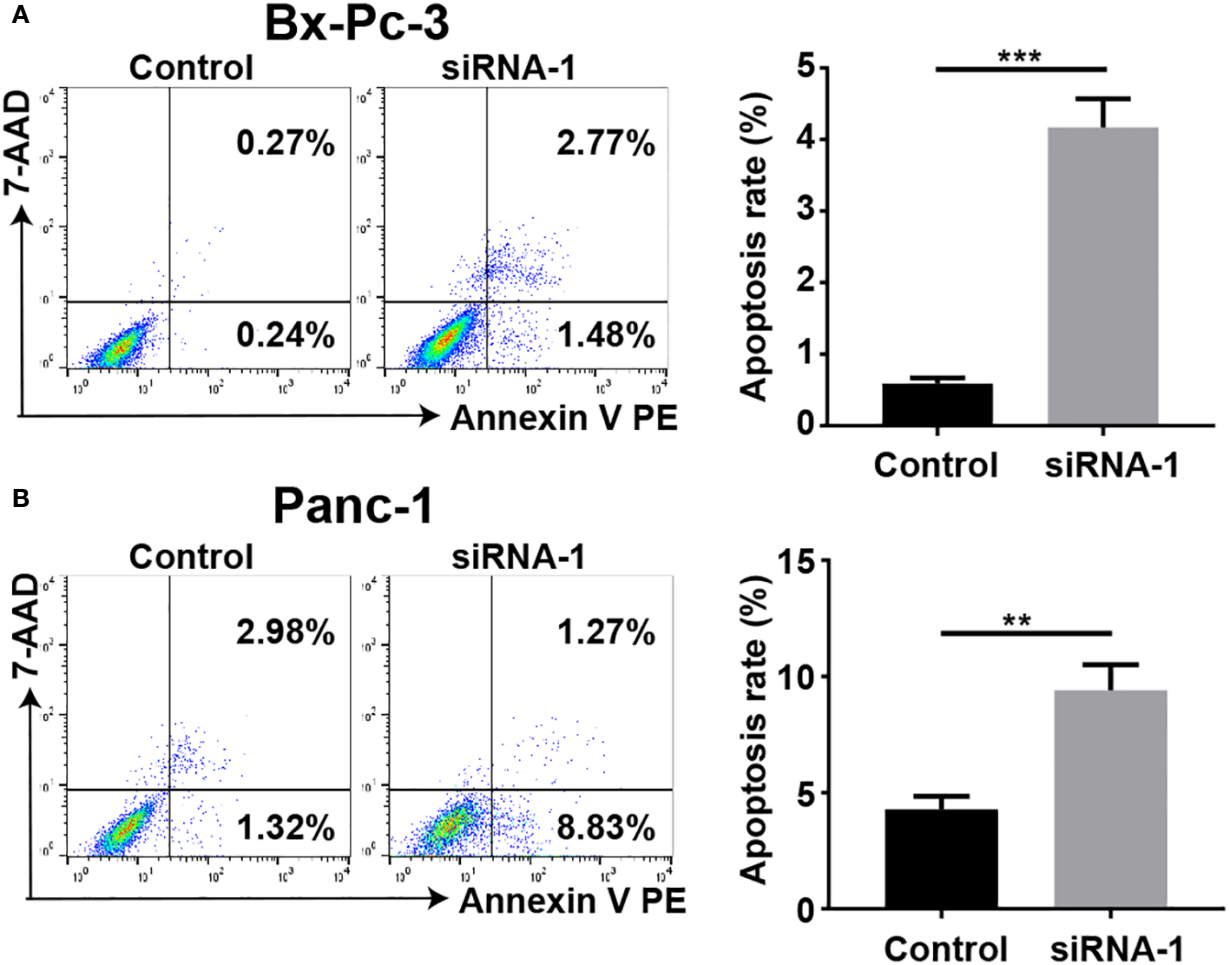
Flow cytometry was employed to determine the apoptosis rate of pancreatic cancer cell lines. **(A)** The apoptotic rate of BXPC-3 significantly increased after PAK2 silencing. **(B)** The apoptotic rate of PANC-1 significantly increased after PAK2 silencing. ** represents P<0.01, *** represents P<0.001.

The results, as shown in the **Figure 12**, demonstrate that upon PAK2 knockdown, the apoptotic rates of BXPC-3 (**Figure 12A**, P < 0.001) and PANC-1 (**Figure 12B**, P < 0.01) cells significantly increased. There is a statistically significant difference between the two groups. Therefore, PAK2 exhibits a significant pro-apoptotic cytotoxic effect.

### 3.13 Detection of TGF-beta pathway-related genes and EMT phenotype-related genes

**Figure 13:**
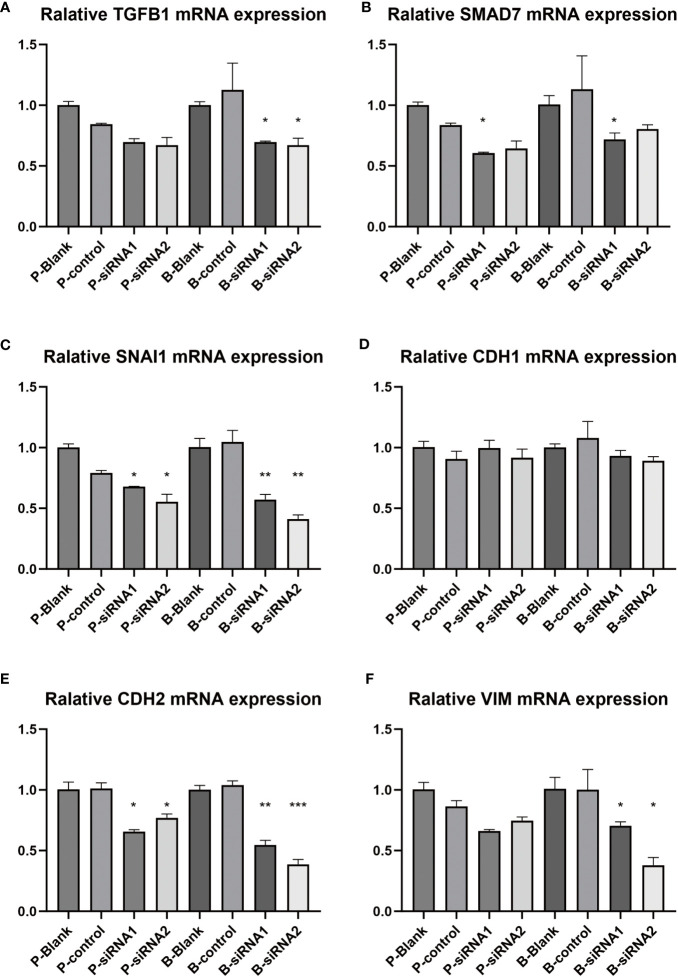
Detection of TGF-beta pathway-related genes and EMT phenotype-related genes. **(A-F)** Real-time fluorescence quantitative PCR was conducted on PANC-1 and Bx-Pc-3 cells transfected with control siRNA and PAK2 siRNAs (n=3, one-way ANOVA, * represents P<0.05, ** represents P<0.01, *** represents P<0.001). **(A)** TGFB1, **(B)** SMAD7, **(C)** SNAI1, **(D)** CDH1, **(E)** CDH2, **(F)** VIM.

In **Figure 13**, we compared the changes in related pathways and phenotype genes after knocking down PAK2 using siRNA. Among them, TGFB1, SMAD7, and SNAI1 are related genes of the TGF-β pathway. TGFB1 is a protein that initiates the TGFβ pathway, and its activation may increase TGFB1. SMAD7 is an inhibitory protein whose expression increases after the TGF-β pathway is activated. SNAI1 is a downstream specific protein whose expression increases after the TGF-β pathway is activated (9–11), and it also has the function of initiating EMT. The three hallmark proteins of EMT activation are CDH1, VIM, and CDH2 (12). Among them, CDH1 expression will be downregulated; VIM and CDH2 expression will be upregulated. As shown in the experimental results, after knocking out PAK2, TGFB1, SMAD7, and SNAI1 all showed a downward trend compared to the control group (**Figures 13A-C**), and at least one siRNA transfection group had a statistically significant decrease, indicating that knocking out PAK2 at least partially weakened the activation of TGF-β. Although the change in CDH1 was not significant between different groups (**Figure 13D**), both VIM and CDH2 showed a significant decrease after knocking down PAK2 (**Figures 13E, F**), indicating that the knockdown of PAK2 affected the progress of EMT. The above experiments indicate that there is a correlation between PAK2 and the TGF-β pathway and EMT phenotype, and in pancreatic cancer, the upregulation of PAK2 may lead to the activation of the TGF-β signaling pathway and EMT.

## 4 Discussion

Pancreatic cancer is an extremely malignant digestive tract tumor. The 2022 cancer statistics reported by Xia et al. reveal that pancreatic cancer constitutes 2.8% of all new cancer cases and contributes to 4.1% of all cancer-related deaths among the 34 types of cancers (13). Hence, pancreatic cancer stands out as one of the most perilous cancers. The insidious and nonspecific nature of early symptoms, combined with its pronounced invasiveness, contributes to frequent occurrences of local tissue invasion and distant metastasis. Consequently, the majority of patients receive a diagnosis at an advanced stage, with only approximately 15-20% of pancreatic cancer patients deemed eligible for early pancreatic resection (3, 14). According to the NCCN guidelines, once distant metastasis occurs, pancreatic cancer is defined as unresectable (4) (15). Given that surgical resection remains the sole potential cure for pancreatic cancer, the prevention and management of distant metastasis emerge as crucial strategies in the clinical treatment of patients with pancreatic cancer (16). According to epidemiological data, pancreatic cancer patients often experience distant metastasis, with the most common sites being the liver, followed by the peritoneum, lungs, pleura, bones, and adrenal glands (17). Furthermore, metastatic pancreatic cancer has been documented in nearly every organ, encompassing the brain, diaphragm, gallbladder, heart, small and large intestines, kidneys, ovaries, pericardium, seminal vesicles, skin, stomach, spleen, testicles, thyroid gland, bladder, and uterus (18). Even patients who have undergone radical pancreatic cancer resection often present with distant metastatic symptoms as a sign of recurrence. Hishinuma et al.’s investigation revealed that approximately three-quarters of patients encountered local recurrence following surgery, half developed liver metastasis, and about one-third experienced peritoneal dissemination (8, 19). In summary, the identification of distant metastasis plays a crucial role in treatment decisions and prognosis assessment for patients with pancreatic cancer. This information is essential for developing personalized treatment plans and early interventions to improve patient survival and quality of life.

Clinical observations indicate that pancreatic cancer exhibits a distinct secondary tumor preference for the liver, which is closely associated with the liver providing a microenvironment favorable for the metastasis of pancreatic cancer cells. The interaction between pancreatic cancer cells and the liver during the process of liver metastasis involves three interconnected stages: firstly, the adhesion of disseminated cancer cells to the liver sinusoids; followed by the early formation of micro-metastases in the hepatic lobules; and finally, the suppression of anti-tumor activity by the immune system (20). The initial step in the liver metastasis of pancreatic cancer involves the adhesion of pancreatic cancer cells to the liver sinusoids. This process is influenced by chemokine-chemokine receptor patterns, as well as the involvement of platelets and neutrophils. Meijer et al. highlighted in their study that CXCL13 plays a significant role in promoting the recruitment of disseminated pancreatic cancer cells expressing CXCR5 to the liver, thereby inducing the growth of liver metastases (21). Moreover, receptor patterns, including CX3CL1/CX3CR1 (22) and CXCR7/CXCR4/CXCL12 (23), have been demonstrated to play crucial roles in the progression of pancreatic cancer liver metastasis. Platelets play a crucial role in the pancreatic cancer liver metastasis process by aggregating to form clots. This clot formation acts as an effective barrier, preventing cancer cells from being cleared by natural killer (NK) cells, monocytes, or macrophages (24). Polymorphonuclear neutrophils (PMN), acting as carriers for cancer cells, possess the capability to directly adhere to the endothelium of hepatic microvessels. Pancreatic cancer cells recruit PMNs through intercellular adhesion molecule-1 (ICAM-1), thereby enhancing the likelihood of cell stagnation in capillaries. Additionally, abnormal expression of the selectin family also contributes to the adhesion of pancreatic cancer cells (25). In the formation stage, pancreatic cancer cells gradually reshape the liver environment by interacting with tumor-associated fibroblasts to create a robust extracellular matrix environment (26, 27), thereby establishing a preliminary hypoxic tumor microenvironment (TME). Hypoxia facilitates the production of vascular endothelial growth factor (VEGF), supporting the formation of new microcirculation (28). Subsequently, pancreatic cancer completes survival and proliferation in the liver sinusoids. In the third stage, pancreatic cancer cells strengthen the gradually established tumor microenvironment, gradually causing the immune killing system to cease by recruiting and transforming other cells in the TME. Suppressed tumor-killing cells including NK cells (29), exhausted T cells (30), and macrophages gradually shift towards the M2 type (31). Immune inhibitory cells including regulatory T cells (Treg) (32) and myeloid-derived suppressor cells (MDSC) infiltrate massively (33), ultimately forming an impregnable immune escape situation.

Due to the rarity of samples from pancreatic cancer liver metastasis and limitations in research techniques, previous studies on pancreatic cancer liver metastasis have mostly been confined to clinical cohort studies. This limitation makes it challenging for researchers to comprehend the microscopic changes occurring during the process of liver metastasis. With the advancements in microarray technology and next-generation sequencing techniques, researchers can explore changes at the gene level during the process of pancreatic cancer liver metastasis by repeatedly analyzing mRNA matrices. However, due to the limitations of sequencing techniques, researchers cannot eliminate the influence of infiltrating cells in sequenced tissues, which has limited the clinical utility of most biomarkers. In recent years, advancements in single-cell sequencing technology have opened up new avenues for studying diseases at the individual cell level. Single-cell sequencing technology provides us with high-resolution gene expression data, allowing for detailed and comprehensive studies of cell gene expression. This is crucial for researching disease-specific cell phenotypes and developing therapeutic approaches. The advancing machine learning algorithms provide powerful tools, accelerating our understanding of biological complexity, propelling advancements in biomedical research, and offering more possibilities for future medical diagnosis and treatment. Therefore, the rise of single-cell sequencing technology combined with machine learning provides us with more comprehensive and in-depth tools for disease research. However, until now, there has been a scarcity of bioinformatics analysis regarding pancreatic cancer liver metastasis, and the methods used in these analyses are often outdated. In this investigation, we undertook a comprehensive analysis of pancreatic cancer liver metastasis utilizing diverse data sources. We utilized single-cell sequencing data to identify transcriptomic changes in epithelial cells as accurately as possible. By combining the advantages of large samples from traditional bulk sequencing results, which allow for prognostic analysis, we obtained satisfactory results. In the selection of key genes, we not only relied on the results of statistical algorithms but also considered the biological significance of genes for selection, supplementing the lack of domain-specific knowledge in unsupervised machine learning. Additionally, we conducted in-depth mechanistic analysis of how PAK2 influences the process of pancreatic cancer liver metastasis and ultimately validated the findings through cell experiments. Therefore, our study not only provides new insights into pancreatic cancer liver metastasis but also offers new research directions for future bioinformatics studies.

P21-activated kinase (PAK) plays a crucial role in regulating a wide range of processes associated with cytoskeletal rearrangement, including cell migration, apoptosis, and cell division (34). P21-activated kinase 2 belongs to the PAK family of serine/threonine kinases and functions as a downstream substrate of Rho family GTPases, including Rac and CDC42 (35). Earlier research has highlighted a significant correlation between P21-activated kinase 2 (PAK2) and the proliferation, adhesion, and migration of various types of tumors (36, 37). There is limited research on the relationship between PAK2 and pancreatic cancer, and existing studies have only demonstrated, at the cellular experimental level, that PAK2 can increase the proliferation and invasive capabilities of pancreatic cancer cells (38). The association of PAK2 with pancreatic cancer hematogenous metastasis was confirmed in our TCGA dataset analysis. Furthermore, based on this, our study proposes that PAK2 is a key gene mediating the liver metastasis of pancreatic cancer. After analyzing other single-cell data, we found that PAK2 has no significant promoting effect on the lung and vaginal metastasis of pancreatic cancer. Therefore, we believe that the promotion of PAK2 in pancreatic cancer liver metastasis is specific. GSVA suggests a strong connection between the overexpression of PAK2 and the activation of the TGF-beta signaling pathway in pancreatic cancer. This association aligns with findings from prior studies (39, 40). The TGF-beta signaling pathway is a classical pro-tumor metastasis pathway, and its activation will further increase the vascular generation ability and epithelial-mesenchymal transition of tumor cells, making the tumor more prone to distant metastasis (41). In our investigation, grouping analysis of cancer cells revealed that elevated PAK2 expression enhances the angiogenesis capability and epithelial-mesenchymal transition of cancer cells, aligning with findings from previous studies. Hence, we have grounds to posit that one of the roles of PAK2 is to foster the liver metastasis of pancreatic cancer through the activation of the TGF-beta signaling pathway. This activation, in turn, enhances the vascular generation ability and epithelial-mesenchymal transition of pancreatic cancer cells. In our proposed temporal sequence analysis, we observed that PAK2 tends to induce cancer cells into a state of lower differentiation, representing a crucial mechanism contributing to the facilitation of liver metastasis in pancreatic cancer.

The tumor microenvironment refers to a highly dynamic integrated network of cellular and non-cellular components formed around tumor cells. This microenvironment encompasses diverse cell types that engage in interactions with tumor cells, including immune cells, endothelial cells, fibroblasts, and interconnected molecular signaling networks (42). The tumor microenvironment is pivotal in orchestrating the dynamics of tumor growth, invasion, and metastasis. Its complex interaction network involves aspects like cytokines, extracellular matrix, angiogenesis, and immune regulation, forming an interconnected ecosystem (43). Tumor cells are vital for maintaining the homeostasis of the tumor microenvironment, and any factor that alters the degree of communication between the tumor and surrounding cells can lead to changes in the homeostasis of the tumor microenvironment, disrupting the anti-tumor immune response or causing stronger immune suppression. In our study, we found that PAK2 is crucial for influencing the communication network of pancreatic cancer cells. The cells with high PAK2 expression receive signals from other cells more frequently, playing a significant role in various signaling networks, directly leading to a significant increase in the strength of multiple signaling networks in liver metastatic lesions. This includes CEACAM, HSPG, and EGF, among others. The CEACAM (carcinoembryonic antigen-related cell adhesion molecule) family, which encodes proteins involved in cell adhesion, plays a crucial role in mediating interactions between tumor cells and surrounding tissues. Specifically, members like CEACAM1 are known to regulate immune responses, potentially influencing the immune microenvironment within tumors through interactions with immune cells. Additionally, the CEACAM protein family is implicated in cell signaling pathways, suggesting potential effects on processes such as cell proliferation, apoptosis, and differentiation (44). Heparan sulfate proteoglycans (HSPGs) are a class of molecules consisting of a core protein covalently linked to heparan sulfate (HS) glycosaminoglycan (GAG) chains. These complex structures are extensively expressed on the cell surface and within the extracellular matrix (ECM), exerting significant influence on various aspects of cellular physiology. HSPGs play crucial roles in processes such as cell proliferation, adhesion, and motility. They are also involved in membrane transport, the formation of extracellular gradients, morphogenesis, and angiogenesis. The diverse functions of HSPGs highlight their importance in regulating fundamental cellular processes and tissue development (45). Epidermal Growth Factor (EGF) is a gene that encodes the epidermal growth factor protein. The EGF protein, upon binding to its receptor, Epidermal Growth Factor Receptor (EGFR), activates downstream signaling pathways, including RAS/MAPK and PI3K/AKT. These pathways play a crucial role in promoting the proliferation of cancer cells. The interaction between EGF and EGFR is a key molecular mechanism that contributes to cell growth and survival, and dysregulation of this pathway is often associated with cancer development and progression (46). Therefore, cells with high expression of PAK2 enhance their proliferative and invasive capabilities by activating these signaling networks, thereby accelerating the occurrence of liver metastasis.

Transcription factors are proteins that play a vital role in gene regulation by binding to specific DNA sequences. This binding interaction enables transcription factors to modulate the transcriptional activity of nearby genes. Transcription factors create complexes with protein-nucleic acid interactions, influencing the regulation of gene activation or suppression. The activity of transcription factors experiences alterations in various cancers through direct mechanisms such as chromosomal translocation, gene amplification or deletion, point mutations, and changes in expression. Furthermore, mutations in non-coding DNA that impact the binding of transcription factors indirectly contribute to these modifications (47). Since single-cell sequencing detects mRNA, the transcription factors that function as proteins have been challenging to analyze at the transcriptome level. However, the DoRothEA package provides a reliable analytical method for interpreting gene expression patterns in single-cell data, inferring the likely activity levels of transcription factors in single cells. In our analysis, we identified numerous transcription factors influenced by PAK2, and after conducting a protein-level analysis, we identified a critical subnetwork among them. In this subnetwork, the NOCA family occupies an important position. The Nuclear Receptor Coactivator (NCOA), also known as the Steroid Receptor Coactivator (SRC) family, comprises proteins primarily responsible for regulating the transcriptional activity of genes through interactions with nuclear receptors. Nuclear receptors, including hormone receptors such as estrogen receptor (ER) and androgen receptor (AR), govern various biological processes in cells. The NCOA family plays a crucial role in the regulation mediated by these nuclear receptors. Research in the context of cancer indicates that abnormal expression of NCOA is closely associated with the occurrence and development of various cancer types (48). In this study, we hypothesize that the NCOA family is implicated in the process of pancreatic cancer liver metastasis regulated by PAK2, although the specific mechanism requires further investigation.

Gemcitabine, as a primary treatment for pancreatic cancer, remains a crucial therapeutic approach in extending the survival of patients. However, the emergence of resistance to gemcitabine significantly diminishes its benefits for individuals with pancreatic cancer (49). In our drug sensitivity prediction, we found that overexpression of PAK2 is associated with decreased sensitivity to gemcitabine treatment. To elucidate the mechanism by which PAK2 induces resistance to gemcitabine, we first performed differential analysis and weighted gene co-expression network analysis between gemcitabine-resistant and sensitive cell lines to identify gemcitabine resistance genes. In pancreatic cancer single-cell data, enrichment analysis revealed a notable enrichment of gemcitabine resistance genes in pancreatic cancer cells. A rank-sum test was conducted to correlate the enrichment scores of gemcitabine resistance gene sets in each cell with the expression level of PAK2. The findings demonstrated a significant correlation between the expression level of PAK2 and the enrichment scores of gemcitabine resistance gene sets Therefore, we have demonstrated that PAK2 can mediate gemcitabine resistance in pancreatic cancer, and genes regulated by PAK2, such as SPRR1B, KRT5, and KRT17, are the major mediators promoting resistance to gemcitabine chemotherapy.

This study conducted a detailed analysis of liver metastasis in pancreatic cancer through the integration of single-cell transcriptomics and bulk sequencing data. However, the study has certain limitations. Due to constraints on the quantity of sequencing data, we were unable to completely eliminate the potential impact of factors such as ethnicity, gender, and age. Furthermore, while we employed standard methods supported by existing literature to obtain the gene set associated with gemcitabine resistance in this study, some genes within the set remain unvalidated, potentially introducing uncertainties into the analysis of gemcitabine resistance. Lastly, although the analysis results of this study were corroborated through cell line experiments, additional evidence from animal experiments, organoid experiments, and large-scale human trials would strengthen our findings. In conclusion, the further development of spatial transcriptomics data and other sequencing technologies may offer deeper insights into the mechanisms underlying PAK2 in the process of pancreatic cancer liver metastasis.

## 5 Conclusion

PAK2 played a pivotal role in promoting the angiogenic capability and epithelial-mesenchymal transition processes of cancer cells by activating the TGF-beta signaling pathway. Simultaneously, it diminished the differentiation level of cancer cells, intensifying their malignancy. Furthermore, PAK2 facilitated communication between cancer cells and cells in the tumor microenvironment, augments cancer cell chemoresistance, and induced alterations in pathways associated with energy metabolism. To sum up, PAK2 emerged as a central gene mediating hepatic metastasis in pancreatic cancer.

## Data availability statement

The original contributions presented in the study are included in the article/**Supplementary Material**. Further inquiries can be directed to the corresponding authors.

## Ethics statement

Ethical approval was not required for the studies on humans in accordance with the local legislation and institutional requirements because only commercially available established cell lines were used.

## Author contributions

HY: Writing – original draft, Writing – review & editing. ZL: Writing – original draft, Writing – review & editing. SZ: Writing – original draft, Writing – review & editing. WW: Writing – review & editing. JZ: Writing – review & editing. DZ: Writing – review & editing. MZ: Writing – review & editing. WZ: Writing – original draft, Writing – review & editing. WX: Writing – original draft, Writing – review & editing. CX: Writing – original draft, Writing – review & editing.

## Supplementary material

Supplementary Figure 1 The flowchart of this study.

## Glossary

**Table:**

hdWGCNA	high-dimensional weighted gene co-expression network analysis
LRG	liver metastasis-related gene
GEO	Gene Expression Omnibus
TCGA	The Cancer Genome Atlas
PCA	principal component analysis
UMAP	Uniform Manifold Approximation and Projection
LASSO	Least Absolute Shrinkage and Selection Operator
SVM-RFE	Support Vector Machine Recursive Feature Elimination
SVM	support vector machines
RF	Random Forest
ROC	Receiver Operating Characteristic
GSVA	Gene Set Variation Analysis
KEGG	Kyoto Encyclopedia of Genes and Genomes
PPI	protein-protein interaction
TF	transcription factor
IC50	half-maximal inhibitory concentration
WGCNA	Weighted Gene Co-expression Network Analysis
AUC	area under the curve
ssGSEA	Single-Sample Gene Set Enrichment Analysis
CopyKAT	Copy Number Karyotyping of Aneuploid Tumors
NK	natural killer cells
PMN	Polymorphonuclear neutrophils
ICAM-1	intercellular adhesion molecule-1
TME	tumor microenvironment
VEGF	vascular endothelial growth factor
Treg	regulatory T cells
MDSC	myeloid-derived suppressor cells
PAK	P21-activated kinase
PAK2	P21-activated kinase 2
CEACAM	carcinoembryonic antigen-related cell adhesion molecule
HSPGs	Heparan sulfate proteoglycans
HS	heparan sulfate
GAG	glycosaminoglycan
ECM	extracellular matrix
EGF	Epidermal Growth Factor
EGFR	Epidermal Growth Factor Receptor
NCOA	Nuclear Receptor Coactivator
ER	estrogen receptor
AR	androgen receptor
