# Supplementary material for: Molecular mechanisms of pancreatic cancer liver metastasis: the role of PAK2
Source: Front Immunol. 2024 Jan 26;15:1347683. doi: 10.3389/fimmu.2024.1347683 (PMC10853442; doi:10.3389/fimmu.2024.1347683)
Supplement: Supplementary file 1 [file Table_1.docx]

Table S1: Database name and website.

| Database | website |
| --- | --- |
| GEO | <https://www.ncbi.nlm.nih.gov/geo/> |
| TCGA | <https://www.cancer.gov/about-nci/organization/ccg/research/structural-genomics/tcga> |
| CellMarker | <http://xteam.xbio.top/CellMarker/> |
| BMC Genome Biology | <https://genomebiology.biomedcentral.com/> |
| The human protein atlas | <https://www.proteinatlas.org/> |
| Cancer Therapeutics Response Portal | <http://portals.broadinstitute.org/ctrp/> |
| Genomics of Drug Sensitivity in Cancer | <https://www.cancerrxgene.org/> |
| Ensemble | <http://asia.ensembl.org/index.html> |
