# Supplementary figures and images for: Molecular mechanisms of pancreatic cancer liver metastasis: the role of PAK2
Source: Front Immunol. 2024 Jan 26;15:1347683. doi: 10.3389/fimmu.2024.1347683 (PMC10853442; doi:10.3389/fimmu.2024.1347683)

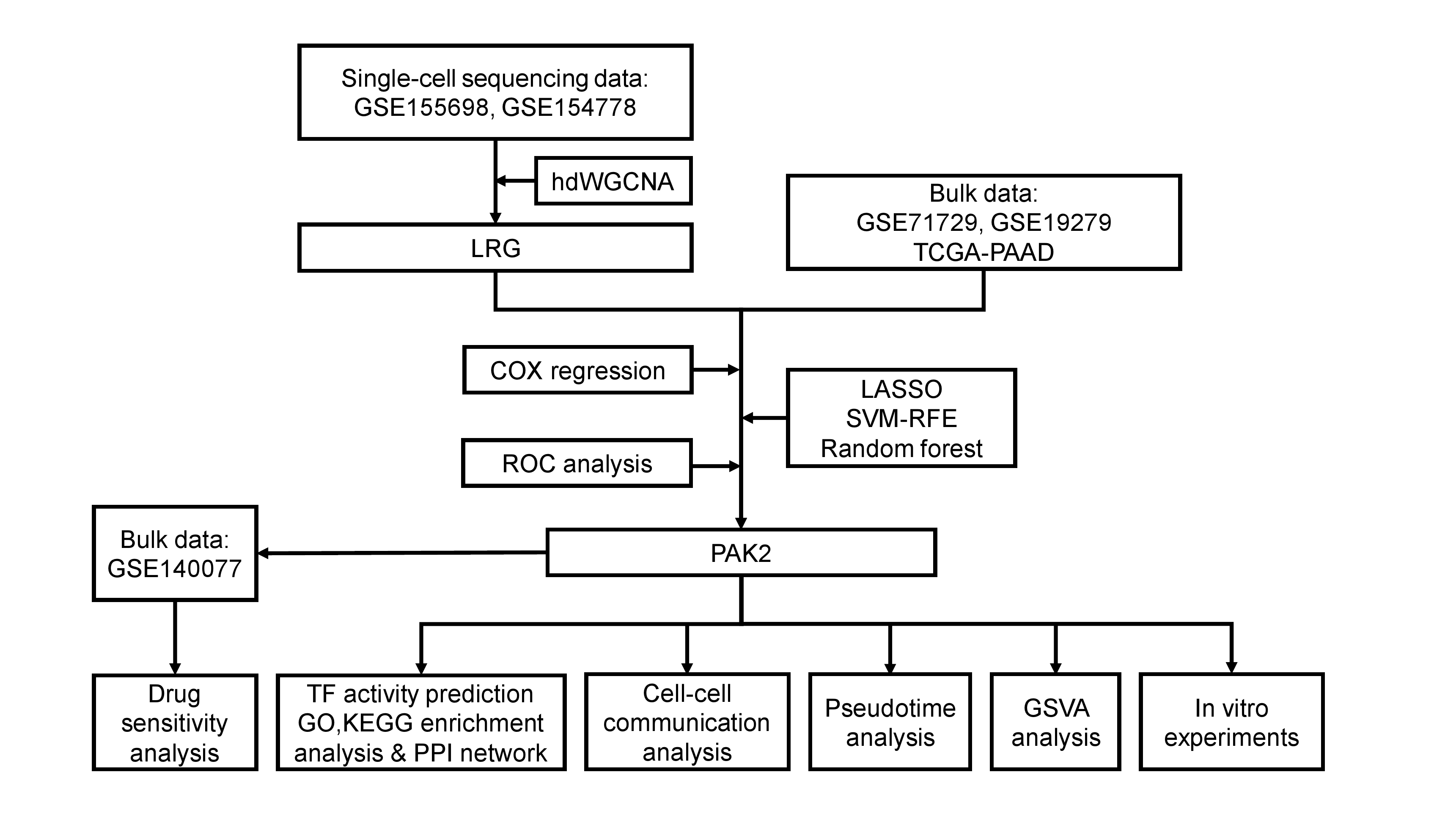

Supplement: Supplementary Figure 1 — The flowchart of this study. [file Image_1.tif]
